# Progressive tauopathy disrupts breathing stability and chemoreflexes during presumptive sleep in mice

**DOI:** 10.3389/fphys.2023.1272980

**Published:** 2023-09-21

**Authors:** Alexandria B. Marciante, Carter Lurk, Luz Mata, Jada Lewis, Leah R. Reznikov, Gordon S. Mitchell

**Affiliations:** ^1^ Breathing Research and Therapeutics Center, Department of Physical Therapy and McKnight Brain Institute, University of Florida, Gainesville, FL, United States; ^2^ Department of Physiological Sciences, University of Florida, Gainesville, FL, United States; ^3^ Center for Translational Research in Neurodegenerative Diseases, Department of Neuroscience, University of Florida, Gainesville, FL, United States

**Keywords:** tauopathy, sleep-disordered breathing, chemoreflex, sleep apnea, plethysmography

## Abstract

**Rationale:** Although sleep apnea occurs in over 50% of individuals with Alzheimer’s Disease (AD) or related tauopathies, little is known concerning the potential role of tauopathy in the pathogenesis of sleep apnea. Here, we tested the hypotheses that, during presumptive sleep, a murine model of tauopathy (rTg4510) exhibits: 1) increased breathing instability; 2) impaired chemoreflex function; and 3) exacerbation of these effects with tauopathy progression.

**Methods:** rTg4510 mice initially develop robust tauopathy in the hippocampus and cortex, and eventually progresses to the brainstem. Type I and II post-sigh apnea, Type III (spontaneous) apnea, sigh, and hypopnea incidence were measured in young adult (5–6 months; n = 10–14/group) and aged (13–15 months; n = 22–24/group) non-transgenic (nTg), monogenic control tetracycline transactivator, and bigenic rTg4510 mice using whole-body plethysmography during presumptive sleep (*i.e.*, eyes closed, curled/laying posture, stable breathing for >200 breaths) while breathing room air (21% O_2_). Peripheral and central chemoreceptor sensitivity were assessed with transient exposures (5 min) to hyperoxia (100% O_2_) or hypercapnia (3% and 5% CO_2_ in 21% O_2_), respectively.

**Results:** We report significant increases in Type I, II, and III apneas (all *p* < 0.001), sighs (*p* = 0.002) and hypopneas (*p* < 0.001) in aged rTg4510 mice, but only Type III apneas in young adult rTg4510 mice (*p* < 0.001) versus age-matched nTg controls. Aged rTg4510 mice exhibited profound chemoreflex impairment versus age matched nTg and tTA mice. In rTg4510 mice, breathing frequency, tidal volume and minute ventilation were not affected by hyperoxic or hypercapnic challenges, in striking contrast to controls. Histological examination revealed hyperphosphorylated tau in brainstem regions involved in the control of breathing (*e.g*., pons, medullary respiratory column, retrotrapezoid nucleus) in aged rTg4510 mice. Neither breathing instability nor hyperphosphorylated tau in brainstem tissues were observed in young adult rTg4510 mice.

**Conclusion:** Older rTg4510 mice exhibit profound impairment in the neural control of breathing, with greater breathing instability and near absence of oxygen and carbon-dioxide chemoreflexes. Breathing impairments paralleled tauopathy progression into brainstem regions that control breathing. These findings are consistent with the idea that tauopathy *per se* undermines chemoreflexes and promotes breathing instability during sleep.

## 1 Introduction

Sleep apnea affects up to one-third of the global population (Lechat et al., 2022). Despite its prevalence, and widespread attention to sleep apnea in recent years, our understanding concerning the pathogenesis of sleep apnea remains incomplete (Dempsey et al., 2010). On one hand, sleep apnea is a major risk factor for development of dementia (Zhu and Zhao, 2018), cognitive decline (Bliwise, 2004; Daulatzai, 2015; Malhotra, 2018; Lajoie et al., 2020; Kam et al., 2022; Lal et al., 2022), and biomarkers for neurodegenerative disease (Yagishita et al., 2017; Carvalho et al., 2020; Kazim et al., 2022; Marciante et al., 2022). On the other hand, central nervous system disorders may directly contribute to the pathogenesis of sleep apnea (Schafer, 2006).

Sleep apnea is observed at far higher rates than the general population in individuals with Alzheimer’s Disease, Parkinson’s Disease, Amyotrophic Lateral Sclerosis, spinal cord injury, stroke, traumatic brain injury, and others (Bliwise, 2004; Bahia and Pereira, 2015; Albdewi et al., 2018; Andrade et al., 2018; Malhotra, 2018). Although the high prevalence in people with acquired brain injury (*e.g.*, traumatic brain injury or stroke) suggests that some element of neurological disease contributes to the pathogenesis of sleep apnea, few investigations have adequately considered the possibility of a bidirectional, causal relationship in any neurological disorder. The fundamental goal of the present study is to begin investigations concerning the possibility that progressive tauopathy directly contributes to the pathogenesis of sleep disordered breathing in a murine model.

To begin investigating the hypothesis that tauopathy causes sleep apnea and dysregulates other key elements of ventilatory control during sleep, we studied the rTg4510 murine model of tauopathy (Ramsden et al., 2005; Santacruz et al., 2005). The rTg4510 mice progressively develop severe tauopathy in hippocampus and cortex, extending to other brain regions with advancing age (Ramsden et al., 2005; Takaichi et al., 2018), including brainstem regions involved in respiratory rhythm generation and chemoreception (Alheid and McCrimmon, 2008). We assessed breathing during presumptive sleep or quiet rest in young adult (5–6 months) and aged (13–15 months) male and female nontransgenic (nTg), monogenic (tTA) and rTg4510 mice to test the hypotheses that tauopathy progression causes age-dependent: 1) irregularities in breathing (including frequent apneas and hypopneas); and 2) diminished peripheral (O_2_) and central (CO_2_) chemoreflexes. We present striking evidence for breathing instability (sighs and apneas) as well as impaired chemoreflex function in aged rTg4510 mice with age-related accumulation of hyperphosphorylated tau in neural circuits generating respiratory rhythm and chemoreflexes.

## 2 Materials and methods

### 2.1 Animals

rTg4510 mice were generated and maintained as previously described (Ramsden et al., 2005; Santacruz et al., 2005). Briefly, the rTg4510 tauopathy model is generated by crossing the parental responder line containing human 0N/4R P301L tau (FVB/N background strain) and the parental activator line, containing the tetracycline transactivator (tTA) gene downstream of the CaMKII promotor (129S6 background strain) (Ramsden et al., 2005; Santacruz et al., 2005). The F1 rTg4510 mice progressively produce human mutant P301L tau primarily in the forebrain, with conditional human tau expression (Ramsden et al., 2005; Santacruz et al., 2005) through the tet-off system (Gossen and Bujard, 1992). Mice were maintained on a standard diet lacking doxycycline to ensure that transgenic tau was expressed throughout their lifetime, reaching 13-fold more human P301L versus endogenous mouse tau protein.

Male and female nontransgenic (nTg), tTA and rTg4510 mice aged to 5–6 months (n = 34) or 13–15 months (n = 70) were used (see Table 1 for group sizes and male/female numbers per group). Up to 5 mice of the same sex were kept in standard mouse cages (29 × 18 × 13 cm) at room temperature (26°C) on a 12-h light-dark cycle (lights on: 07:00–19:00 h) with *ad libitum* access to food and water. Mice were cared for in accordance with guidelines published in the Guide for the Care and Use of Laboratory Animals (eighth Ed., 2011), the National Institutes of Health and American Association for Laboratory Animal Science guidelines. All procedures involving live mice received prior approval from the Institutional Animal Care and Use Committee of the University of Florida.

**TABLE 1:** Body mass (grams, g) and rectal temperature (degrees Celsius, °C) of all young and old mice.

*Groups*	*n’s*	*Body Weight (g)*	*Body Temp. (˚C)*
***Young (5–6 mon)***			
nonTg	10 (4)	30.1 ± 1.3	37.3 ± 0.2
tTA	10 (6)	32.9 ± 1.5	37.3 ± 0.1
rTg4510	14 (7)	28.8 ± 0.8	**36.8 ± 0.2^a^**
***Old (13–15 mon)***			
nonTg	24 (7)	34.7 ± 1.0	**36.8 ± 0.1^b^**
tTA	24 (13)	36.8 ± 0.9	**36.6 ± 0.1^b^**
rTg4510	22 (12)	**26.2 ± 0.7^a^**	36.5 ± 0.2

Data are presented as mean ± SEM.

Group n’s are represented as: total # of mice in a group (# of males). Significant values are bolded.

^a^ *p* < 0.010, significant difference from age-matched nTg.

^b^ *p* < 0.020, significant difference from young-matched genotype.

### 2.2 Whole-body plethysmography in conscious mice

Whole-body mouse plethysmography chambers (200 mL) were ventilated continuously with 21% oxygen (balance nitrogen; 500 mL/min). Volume calibration was performed during each measurement session by injecting a known volume of air (10 mL) into the chamber. Prior to collecting data, mice underwent 3 acclimation sessions ∼2 h long in the plethysmograph chamber over 3 days. On the fourth day, ventilation was measured during a quiet resting state in ambient air via whole body plethysmography (SCIREQ Scientific Respiratory Equipment Inc., Montréal, Canada). To establish baseline conditions, mice were allowed ∼30 min to acclimate to the chamber before measurements were made over ∼1 h.

A subset of aged mice were also exposed to 5 min of 100% inspired O_2_ and to hypercapnia (3% and 5% CO_2_) at the end of recording periods to assess peripheral (100% O_2_) and CO_2_ chemoreflex responses. Prior to recording, mice were weighed and rectal temperature measured with a thermistor (Model TC-1000 Temperature Controller, CWE, United States) to calculate breathing volumes. Plethysmography recording sessions were video recorded to align breathing patterns with mouse activity state.

### 2.3 Plethysmography analysis

EMKA Technologies IOX software v2.10.0.40 (Paris, France) was used to detect individual breaths during quiet rest and analyzed for respiratory rate (f_R_; breaths per minute), tidal volume (V_T_; microliters per Gram), minute volume (V_E_; microliters per min per Gram), expiratory time (T_E_; milliseconds), inspiratory time (T_I_; milliseconds) and total breathing cycle time (T_Total_). Intervals consisting of at least 100 breaths during quiet rest were analyzed for each genotype in each age group (young: 5–6 months old; aged: 13–15 months old) during normoxia and hyperoxia (100% O_2_), and hypercapnia (3% and 5% inspired CO_2_) in a subset of aged mice. Measurements from all intervals during quiet breathing from a single mouse recording session were then averaged together and represent the average for a single mouse (*n*). Quiet resting breaths were defined by breath waveform regularity and by individual mouse activity state as assessed by video recording (eyes closed, hunched stature/curled). An irregularity score (ABS [(T_n_–T_(n–1)_)/T_(n–1)_]*100) was calculated (Viemari et al., 2011; Sheikhbahaei et al., 2017). Only periods in which the animal is at rest were used for analysis; breaths during activity were excluded from analyses (Jensen et al., 2019).

Sighs, post-sigh apneas (Type I-III), and hypopneas were assessed in the analyzed breath periods. We report sigh frequency (sighs per min), sigh inspiratory time (sT_I_), and sigh amplitude. For post-sigh and spontaneous apneas and hypopneas, we report their frequency (per min). An apnea was defined as a cessation in breathing lasting at least 2 average respiratory cycles (end expiratory pause; EEP) or more than 40 ms. For raw breathing traces, inspiration is negative and expiration is positive; hypopnea was defined as a ≥30% reduction in tidal volume from the average trend for at least 200 ms (Peng et al., 2017); 2) part of the breath during expiration crossed the zero line; and 3) the EEP for each breath was less than 20 ms.

Finally, we also report Penh (enhanced pause). Penh is an arbitrary, dimensionless measure calculated as Pause*(Post-Expiratory Pause/Post-Inspiratory Pause); Penh is suggested to be an indicator of bronchoconstriction (SCIREQ Scientific Respiratory Equipment Inc., Montréal, Canada). Increased bronchoconstriction is considered to be paralleled with an increase in Penh. Experiments were blinded to mouse genotype.

### 2.4 FlexiVent in anesthetized mice

FlexiVent procedures were performed as previously described (Reznikov et al., 2016; Reznikov et al., 2018a) on a subset of 13–15 month-old nTg, tTA or rTg4510 mice (n = 34). Briefly, a tracheotomy was performed in anesthetized mice (ketamine/xylazine/acepromazine) and a cannula (blunted 18 g needle) inserted into the trachea. Mice were ventilated at 150 breaths/min with a volume of 10 mL/kg body mass and administered rocuronium bromide. Increasing doses of methacholine were aerosolized using an ultrasonic nebulizer. The aerosols were delivered for 10 s into the inspiratory line of the ventilator. Measurements for each methacholine dose were taken at 10 s intervals over the course of 3.5 min.

### 2.5 Mouse histological and immunohistochemical analysis

Young and aged rTg4510 mice (n = 3 each group) were euthanized by an overdose of isoflurane followed by transcardial perfusion with ice-cold PBS. Left brains were fixed in 10% formalin for 48–72 h before being switched to PBS, processed, embedded in paraffin, and cut into 5 μm sagittal sections. Hematoxylin and Eosin (H&E) staining were performed on sample brain sections to guide brain alignment of blank sections according to the Mouse Brain Atlas (Paxinos, 2001). Experimental slides were used within the range of 0.60–1.20 mm lateral to the midline. After alignment, 5 brain sections per mouse were used for AT-100 (mouse anti-human pT212/S214 tau IgG; ThermoFisher, #MN1060) staining, with a minimum distance of 15 µm between sections from the same animal for each stain. Tau antibody IHC staining was conducted at Dr. Dennis Dickson’s lab at the Mayo Clinic (Jacksonville, FL) using an auto-stainer. Briefly, the tissue was deparaffinized in xylene and rehydrated in decreasing concentrations of ethanol (100%, 95% and 70%) before washing in deionized water. Sections were then heated in deionized water for 30 min for antigen retrieval. After retrieval, tissue slides were placed into the auto-stainer and an antibody specific IHC protocol program was run. Immunohistology was performed with AT-100 (pT212/S214) antibody (1:1,000) to detect hyperphosphorylated tau as previously described (Ramsden et al., 2005; Santacruz et al., 2005). Slides were digitally scanned using the Aperio ScanScope XT (Leica Biosystems) image scanner and viewed in ImageScope.

Neither brainstem nor midbrain from young rTg4510 and from young and aged control mice exhibited any accumulation of hyperphosphorylated tau [data not shown; *see* (Ramsden et al., 2005)]. Consequently, statistical analysis of AT-100 expression in young versus aged rTg4510 mice is not reported (Dutschmann et al., 2010). Images (1,516 × 905 pixels) from aged rTg4510 mice were aligned to the reference brain section image in the Mouse Brain Atlas (Paxinos, 2001) and a heat map for AT-100 staining was created as previously described (Marciante et al., 2020) using ImageJ (NIH). To create the heat map, the images were converted to 8-bit grayscale images. The pixel gray values (0–255) were measured for all images. Each of the 1,516 × 905 pixels were used to create a heat map representing average hyperphosphorylated tau (AT-100) levels in the brainstem and midbrain among aged rTg4510 mice. The heat map was overlaid on the reference brain section image to estimate distribution of abnormally phosphorylated tau in brainstem and midbrain.

### 2.6 Statistical analysis

A two-way mixed effects ANOVA was performed for plethysmography variables with age as a repeated measure and genotype as a main factor. *Post-hoc* comparisons were performed using a Holm-Sidak multiple comparisons test; major comparisons were focused on age effects within genotype and versus age-matched nontransgenic (nTg) control mice. A two-way ANOVA was performed for FlexiVent studies with methacholine dose as a repeated measure, and genotype as a main factor. *Post-hoc* comparisons were performed using a Holm-Sidak multiple comparisons test; major comparisons were focused on effect of genotype versus nontransgenic (nTg) control mice. Significance was assigned as *p* < 0.05. All statistical analyses were performed using GraphPad Prism 9.0a or Sigmaplot (v12). Data are presented as mean ± standard error of the mean (SEM).

## 3 Results

### 3.1 Body weight and temperature measurements

Prior to each plethysmography recording session, mice were weighed and rectal temperature measured to calculate ventilation (Table 1). Although there was no genotype effect on body mass between groups in young mice (F_2,31_ = 3.302, *p* = 0.050; one-way ANOVA), genotype had a significant effect on body mass in old mice (F_2,67_ = 41.297, *p* < 0.001), with old rTg4510 mice weighing less than age-matched nonTg controls (*p* < 0.001; Holm-Sidak *post hoc*). Body temperature measurements for young mice were significantly influenced by genotype (F_2,31_ = 5.725, *p* = 0.008); young rT4510 mice had lower body temperatures versus age-matched controls (*p* = 0.012). Although there was no significant effect of genotype on body temperature in aged mice (F_2,67_ = 1.104, *p* = 0.337), body temperature was lower in older versus younger nTg (*p* = 0.016) and tTA mice (*p* = 0.006).

### 3.2 Breathing in old rTg4510 mice

#### 3.2.1 Breathing stability/irregularity

**FIGURE 1:**
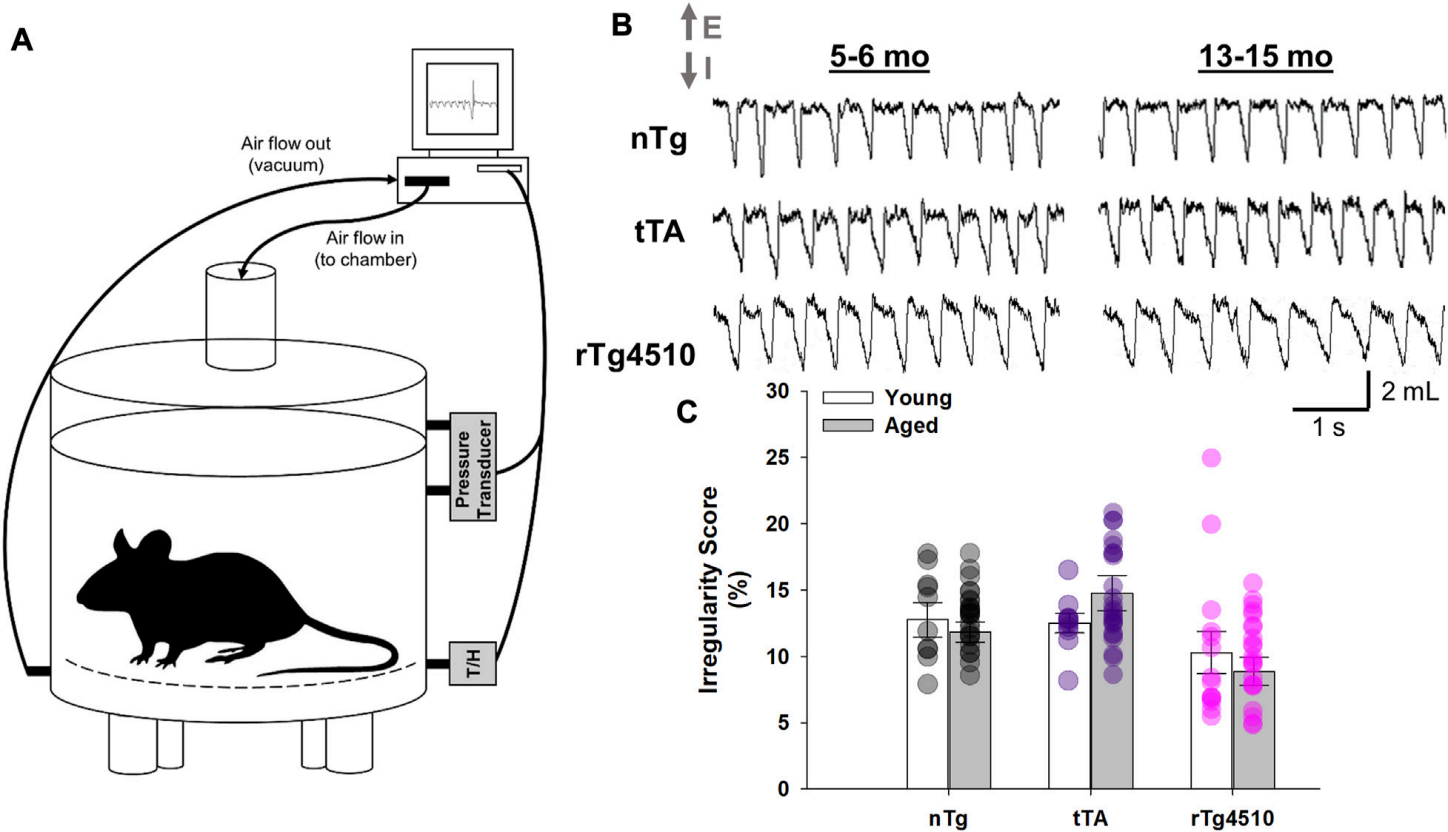
Whole-body plethysmography recordings in unrestrained, conscious nontransgenic (nTg), monogenic tTA, and rTg4510 mice. **(A)** Schematic representation of the experimental setup used to record breathing in unrestrained, conscious mouse placed in a 200 mL recording chamber. **(B)** Individual traces show spirograms (E, expiration; I, inspiration) recorded from young adult (5–6 month old; left column) and aged (13–15 month old; right column) nTg, tTA and rTg4510 mice placed in ambient conditions. **(C)** There was no significant difference in the average 100-breath scores for breathing irregularity during quiet rest between young and old nTg (black circles), tTA (purple circles) and rTg4510 (pink circles) mice (*p* = 0.062). Data presented as mean ± SEM.

The rTg4510 transgenic mouse develops age-dependent tau pathology, with pre-tangle pathology appearing at ∼1.5 months and neurofibrillary tangles at ∼2.5 months in hippocampus and cortex, respectively; it then progresses to other brain regions, such as the limbic system, with advancing age (Ramsden et al., 2005). Here, we analyzed breathing in unrestrained, conscious young (5–6 months) and old (13–15 months) nTg, tTA and rTg4510 mice during presumptive sleep or “quiet rest” (*i.e.*, inactive, eyes closed, curled/laying posture, stable breathing for >200 breaths; Figures 1A, B) (Dutschmann et al., 2010; Jensen et al., 2019) when breathing air in a standard whole-body plethysmograph (Jacky, 1978). One-hundred breath intervals were analyzed during stable breathing periods. Irregularity scores for the 100-breath intervals were calculated for young and aged mice to characterize breathing between age and genotype (Figure 1C); there was no effect of age or genotype on average irregularity scores for 100 breath intervals (F_2,103_ = 2.854, *p* = 0.062; two-way mixed effects ANOVA).

#### 3.2.2 Breath timing

**FIGURE 2:**
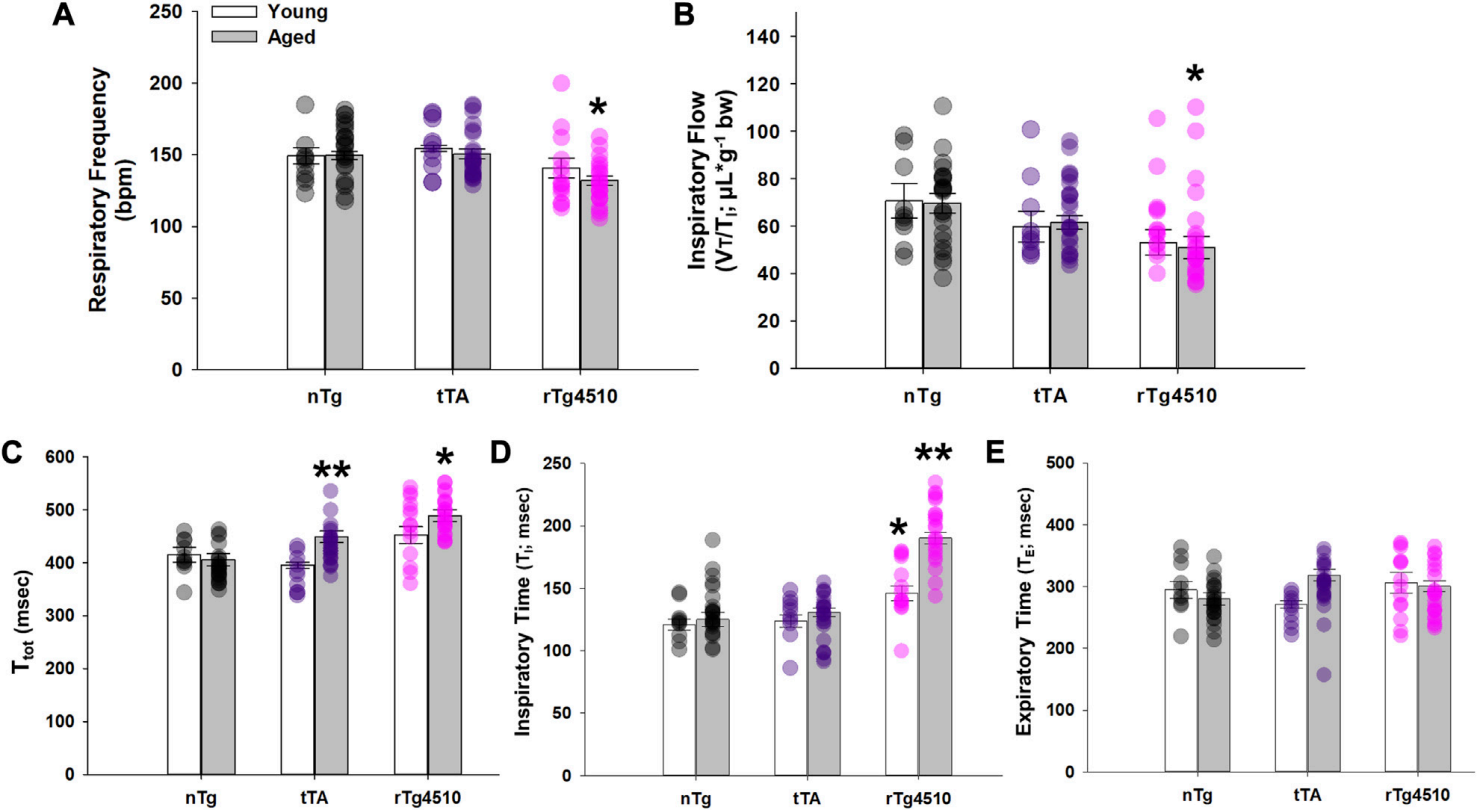
Respiratory cycle timing of nontransgenic (nTg), monogenic tTA, and rTg4510 mice during presumptive sleep. Respiratory cycle timing of young adult (white bars) and aged (gray bars) nTg, tTA and rTg4510 mice shown for **(A)** respiratory frequency in breaths per minute (bpm); **(B)** respiratory drive (V_T_/T_i_); **(C)** total time (T_total_); **(D)** inspiratory time (T_i_); **(E)** and expiratory time (T_e_). *, *p* < 0.050 vs*.* age-matched nTg control; **, *p* < 0.050 vs*.* all comparisons. Data presented as mean ± SEM.

In young mice, there was no difference in respiratory frequency (f_R_; breaths per minute, bpm; Figure 2A) between tTA (155 ± 2 bpm; *p* = 0.481) or rTg4510 (141 ± 7; *p* = 0.387) versus nTg mice (149 ± 6 bpm). In contrast, f_R_ of aged rTg4510 (132 ± 3 bpm; *p* = 0.001) was significantly reduced versus age-matched nTg mice (150 ± 3). There was no difference between old nTg or tTA mice (151 ± 3; *p* = 0.831). A similar trend was also apparent in mean inspiratory flow, V_T_/T_I_ (in µL/g; Figure 2B), with no differences between young versus nTg mice (nTg: 70.7 ± 7.4; tTA: 59.7 ± 6.4, *p* = 0.220; rTg4510: 53.0 ± 5.3, *p* = 0.068). Aged rTg4510 had significantly reduced V_T_/T_I_ versus aged nTg mice (nTg: 69.7 ± 4.2; tTA: 61.5 ± 3.0, *p* = 0.159; rTg4510: 50.9 ± 4.6, *p* = 0.004).

In agreement with respiratory frequency data, total respiratory cycle time (T_tot_) was significantly longer in aged rTg4510 (489 ± 11; ms) vs*.* nTg mice (406 ± 12; *p* < 0.001; Figure 2C). Aged tTA mice also had a modest yet significantly longer T_tot_ vs*.* nTg mice (449 ± 11 ms; *p* = 0.041). There was no difference in T_tot_ between any genotype in young age mice (all *p* < 0.050). Although there was a significant interaction between age and genotype on inspiratory time (T_I_; F_2,103_ = 10.733; *p* < 0.001; Figure 2D), T_I_ was significantly greater in young adult (146 ± 6 ms) and aged (190 ± 5 ms) rTg4510 mice vs*.* age-matched nTg mice (*young:* 121 ± 4 ms, *p* = 0.004; *aged*: 125 ± 6 ms, *p* < 0.001). T_I_ also significantly increased in aged rTg4510 mice (*p* < 0.001). There was no significant interaction between age and genotype on expiratory time (T_E_; F_2,103_ = 3.037; *p* = 0.053; Figure 2E).

#### 3.2.3 Tidal volume (V_T_)

**FIGURE 3:**
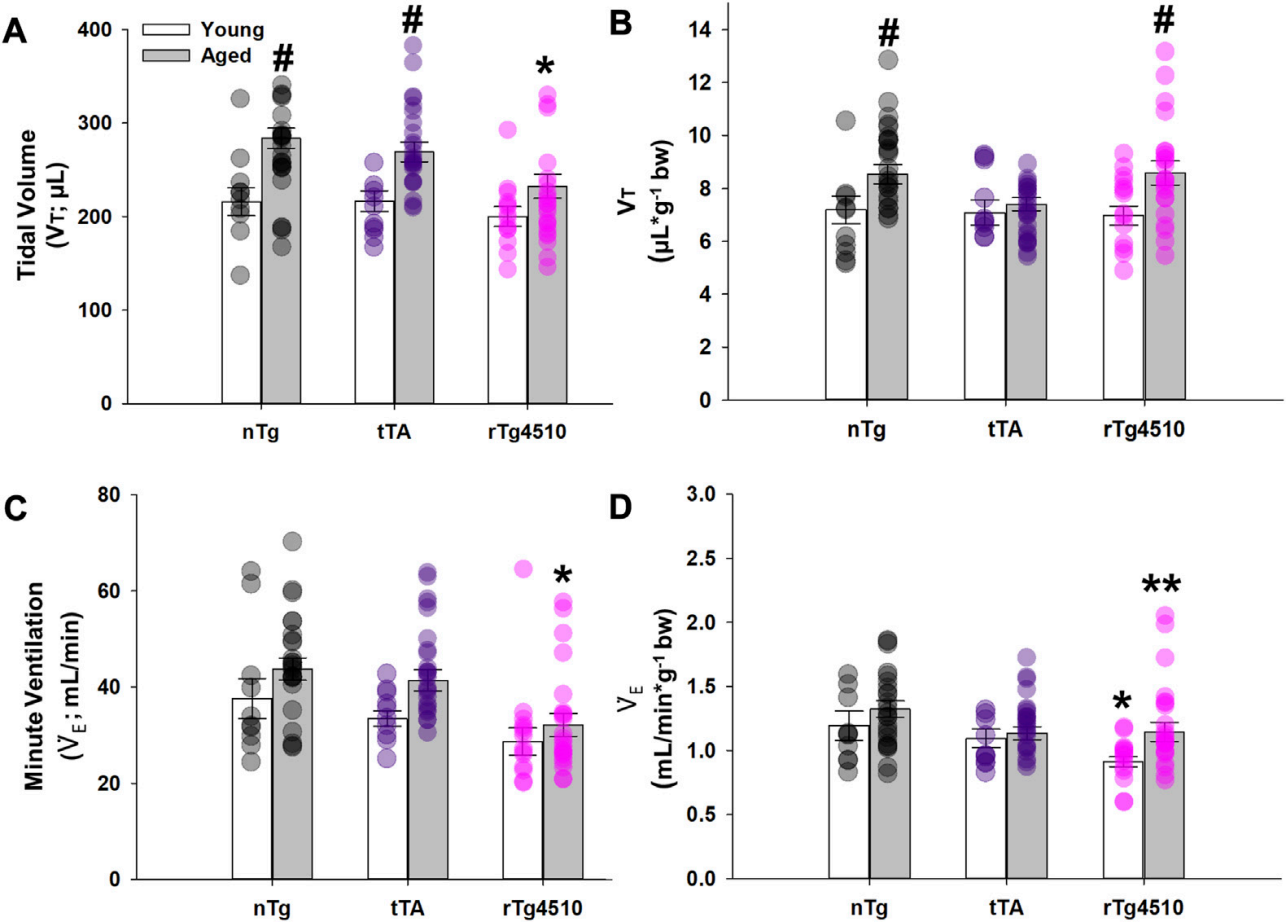
Baseline breathing characteristics of nontransgenic (nTg), monogenic tTA, and rTg4510 mice during presumptive sleep. Baseline breathing measurements of young adult (white bars) and aged (gray bars) nTg, tTA and rTg4510 mice, including: **(A)** tidal volume (V_T_); **(B)** V_T_ normalized to body mass; **(C)** minute ventilation ( $\dot{V}$ _E_); and **(D)** $\dot{V}$ _E_ normalized to body mass. #, *p* < 0.050 vs*.* young corresponding genotype; *, *p* < 0.050 vs*.* age-matched nTg control; **, *p* < 0.050 vs*.* all comparisons. Data presented as mean ± SEM.

The volume of air inspired/expired per breath, normally increases with body mass from birth to old-age in normal mice (Lutfi, 2017). However, the aged rTg4510 mice studied here have a lower body mass *versus* age-matched nTg controls due to disease progression (Table 1). Since it is unclear if V_T_ and minute ventilation (V_E_, in mL/min) should be normalized to body mass, these variables are presented in both non-normalized (Figures 3A, C) and normalized forms (Figures 3B, D).

Non-normalized values of V_T_ (µL) significantly increased with age (F_1,98_ = 22.336; *p* < 0.001) and varied by genotype (F_2,98_ = 3.817, *p* = 0.025); the genotype effect was due exclusively to nTg (young: 216.0 ± 15.0 µL; old: 283.6 ± 10.8 µL; *p* < 0.001) and tTa (young: 216.4 ± 11.1 µL; old: 269.1 ± 10.6 µL; *p* = 0.007) mice (Figure 3A). In contrast, V_T_ in rTg4510 mice did not change with age (young: 200.0 ± 10.5 µL; old: 232.5 ± 13.0 µL; *p* = 0.066), and was significantly lower than in age-matched nTg mice (*p* = 0.002). When V_T_ was normalized to body mass, age (F_1,98_ = 9.741, *p* = 0.002) had a significant effect on V_T_ (µL/g, bw), regardless of genotype (F_2,98_ = 1.177, *p* = 0.313; Figure 3B). In V_T_, there was no statistically significant age × genotype interaction.

#### 3.2.4 Minute ventilation ( $\dot{V}$ _E_; mL/min)

Non-normalized $\dot{V}$ _E_ was not different in young versus aged nTg, tTA or rTg4510 mice (*p* = 0.136, *p* = 0.054 and *p* = 0.352, respectively; Figure 3C); however, $\dot{V}$ _E_ was significantly lower in aged rTg4510 versus nTg mice (*p* < 0.001). When normalized to body mass, $\dot{V}$ _E_ was significantly greater for aged vs*.* young adult rTg4510 mice (*p* = 0.021), but $\dot{V}$ _E_ of both young and aged rTg4510 mice was significantly lower vs*.* age-matched nTg mice (*p* = 0.042 and *p* = 0.040, respectively; Figure 3D).

### 3.3 Age-dependent progression of abnormal breathing patterns in rTg4510 mice

**FIGURE 4:**
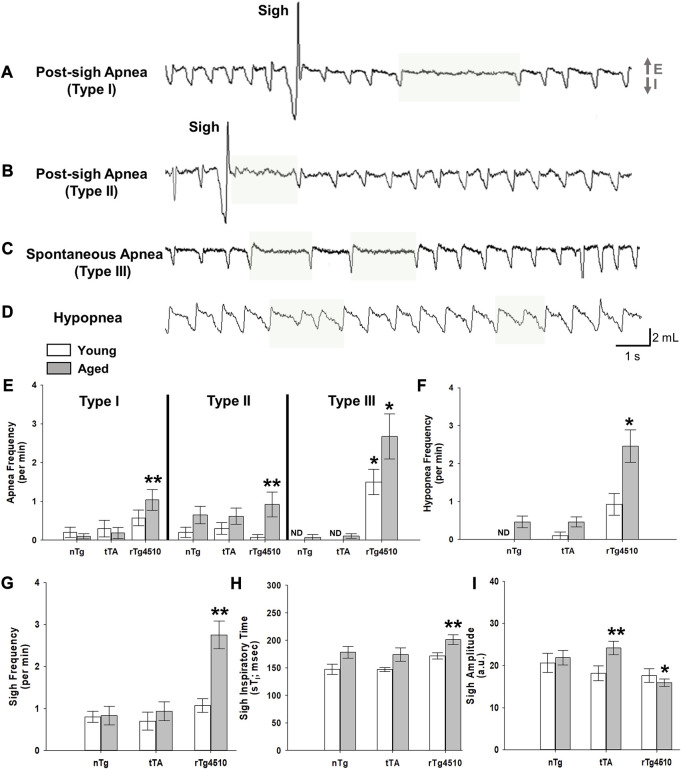
Sigh, apnea and hypopnea frequencies are elevated with advanced tauopathy in aged rTg4510 mice. Representative plethysmograph traces of detected breathe disturbances (E, expiration; I, inspiration) are highlighted for **(A) (A)** Type I post-sigh apnea, or an apnea that occurs several breaths after a sigh or augmented breath; **(B)** Type II post-sigh apnea, or an apnea occurring immediately following a sigh or augmented breath; **(C)** Type III spontaneous apnea; **(D)** hypopnea, or period of shallowing breathing. In **(E)**, apnea frequency is shown for young adult (white bars) and aged (gray bars) nTg, tTA and rTg4510 mice during presumptive sleep. For all apnea types, aged rTg4510 mice experienced the greatest number of incidences per minute versus nTg controls (all *p* < 0.050). Young rTg4510 mice experienced significantly more Type III spontaneous apnea events versus controls (*p* < 0.001). In **(F, G)**, aged rTg4510 mice experienced significantly more hypopnea and sigh events per minute, respectively, versus controls (both *p* < 0.001). In **(H)**, inspiratory time during a sigh (sT_i_) was significantly longer for aged rTg4510 mice versus young rTg4510 (*p* = 0.026) and age-matched nTg control mice (*p* = 0.049). Despite a longer sT_i_, sigh amplitude **(I)** was smaller in aged rTg4510 mice versus age-matched nTg controls (*p* < 0.001). *, *p* < 0.050 vs*.* age-matched nTg control; **, *p* < 0.050 vs*.* all comparisons. Data presented as mean ± SEM.

Apneas and sigh frequencies (per min) were measured for young and old nTg, tTA and rTg4510 mice during presumptive sleep. Apneic events were defined as a cessation of respiratory flow of more than two average respiratory cycles (Nakamura et al., 2003). Apneas varied in duration from 0.9 to 4.0 s, corresponding to 2.1 to 8.8 respiratory cycle times immediately prior to the apnea. We adopted definitions for different types of apneas as defined previously (Yamauchi et al., 2008; Peng et al., 2017). Type I post-sigh apneas are apneas that occur after a sigh with a delay of several normal breaths (Figure 4A). A Type II post-sigh apnea occurs immediately following a sigh without delay (Figure 4B) (Yamauchi et al., 2008). Type III apneas are spontaneous apneas during normal breathing (Figure 4C) (Yamauchi et al., 2008; Peng et al., 2017). Hypopneas are defined as periods of shallow or slow breathing with ≥30% reduction in tidal volume from baseline levels (Peng et al., 2017); hypopneas varied in duration from 1.0 to 3.0 s (Figure 4D).

During presumptive sleep, there was a marginally significant interaction of genotype and age on Type I post-sigh apnea frequency (F_2,64_ = 3.113, *p* = 0.050, 2-way mixed effects ANOVA), driven by a significantly higher sigh frequency in aged rTg4510 mice (1.1 ± 0.3 apneas/min) vs*.* young (0.6 ± 0.2; *p* = 0.019) and age-matched nTg controls (0.1 ± 0.1; *p* < 0.001; Figure 4E). Genotype and age also affected Type II post-sigh apnea frequency (F_2,31_ = 6.318, *p* = 0.005). Sigh frequency was significantly higher in aged rTg4510 (0.9 ± 0.3/min) vs*.* young (0.1 ± 0.1; *p* < 0.001) and age-matched nTg controls (0.7 ± 0.2; *p* = 0.001; Figure 4E). Perhaps the most striking result is the incidence of Type III spontaneous apneas, where genotype (F_2,64_ = 42.253, *p* < 0.001) and age (F_1,54_ = 7.069, *p* = 0.010) significantly influenced apnea frequency (Figure 4E). In young and aged rTg4510 mice (1.5 ± 0.3/min and 2.7 ± 0.6, respectively; both *p* < 0.001 vs*.* age-matched nTg controls). Hypopnea frequency was also significantly influenced by genotype (F_2,47_ = 19.840, *p* < 0.001); aged rTg4510 exhibited a significantly greater rate of hypopneas (2.5 ± 0.4) vs*.* aged-matched nTg mice (0.5 ± 0.2; *p* < 0.001; Figure 4F). There were no differences in apnea or hypopnea frequency for tTA versus nTg mice for either young or aged mice (all *p* > 0.050).

Sigh frequency during presumptive sleep was also affected by genotype (F_1,98_ = 16.110, *p* < 0.001) and age (F_1,98_ = 4.448, *p* = 0.038), with an increased sigh frequency in aged rTg4510 (2.8 ± 0.3/min) vs*.* nTg mice (0.8 ± 0.2; *p* < 0.001; Figure 4G). Aged rTg4510 mice also had a significantly longer T_I_ values during sighs (201.3 ± 8.0 ms) vs*.* young rTg4510 (171.5 ± 10.3 m; *p* = 0.026) or age-matched nTg mice (178.2 ± 9.6 ms; *p* = 0.049; Figure 4H), but a smaller sigh amplitude (15.9 ± 1.2 a.u.) vs*.* age-matched nTg mice (21.8 ± 1.1 a.u.; *p* < 0.001; Figure 4I).

### 3.4 Abolished peripheral chemoreflex in aged rTg4510 mice

**FIGURE 5:**
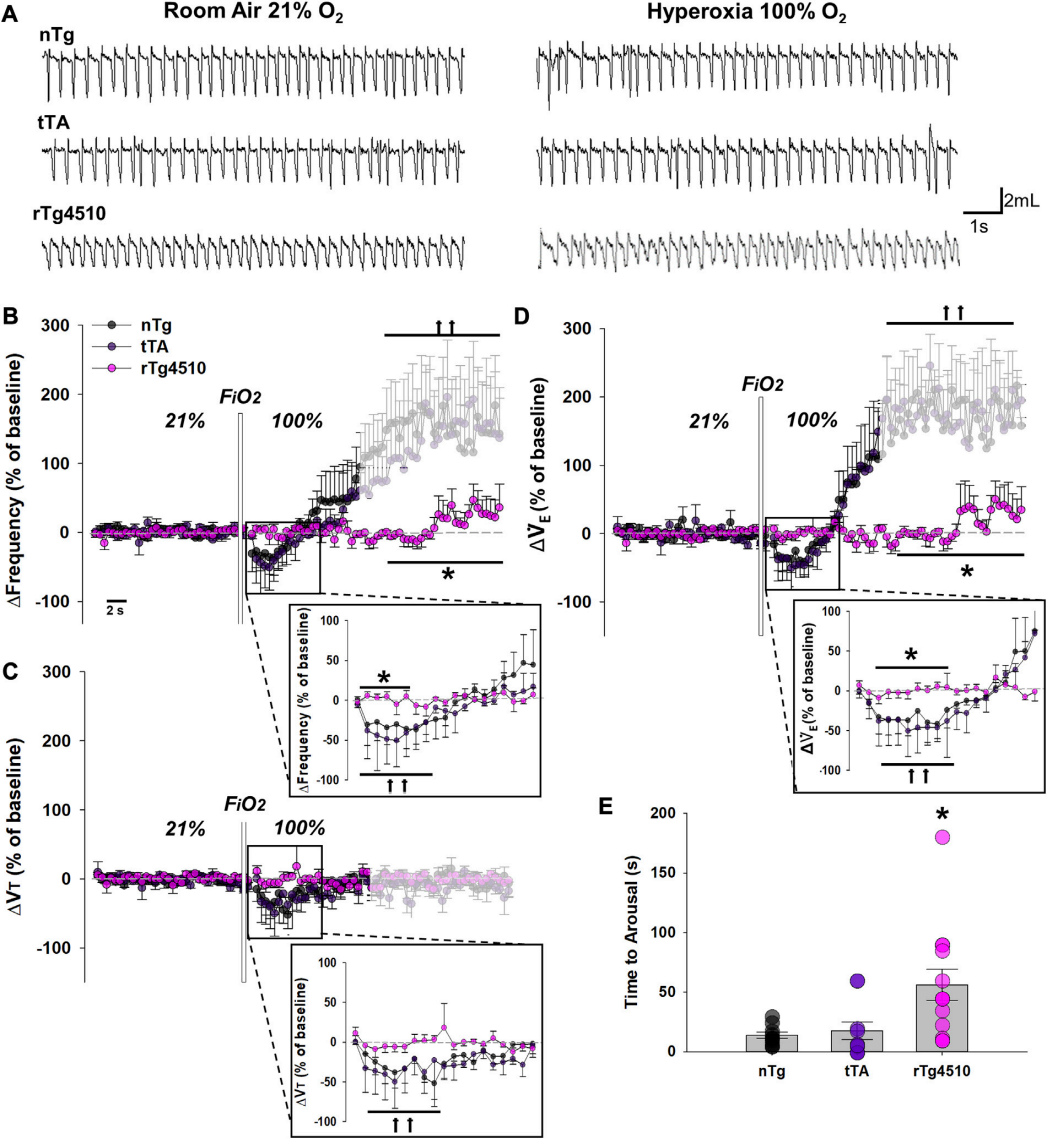
The peripheral chemoreflex is abolished in aged rTg4510 mice during 100% O_2_ hyperoxic challenge. In **(A)**, representative plethysmograph traces of stable breathing during presumptive sleep to room air (21% O_2_; left column) and hyperoxia (100% O_2_; right column) in aged nTg (top), tTA (middle), and rTg4510 (bottom) mice. Aged rTg4510 mice did not change their respiratory frequency **(B)**, tidal volume (V_T_), **(C)**, or minute ventilation ( $\dot{V}$ _E_), **(D)** in response to hyperoxia. In **(E)**, aged nTg and tTa mice were aroused from presumptive sleep in ∼20 s following onset of hyperoxic challenge, while aged rTg4510 mice did not become aroused until ∼60 s *, *p* < 0.050 vs*.* age-matched nTg control; ††, *p* < 0.050 vs*.* baseline. Data presented as mean ± SEM.

Mice were exposed to hyperoxia (100% O_2_) for 5 min to test peripheral chemoreflex function (Figure 5A). Changes in f_R_ (Figure 5B), V_T_ (Figure 5C) and $\dot{V}$ _E_ (Figure 5D) from baseline were analyzed in 2 segments: 1) immediately following the onset of hyperoxia until mice aroused from quiet rest/presumptive sleep, and 2) time during hyperoxia following arousal. With respect to f_R_, there was a significant interaction between genotype and time in hyperoxia (F_4,74_ = 6.801, *p* < 0.001; 2-way RM ANOVA; Figure 5B). Immediately following hyperoxia onset, aged nTg and tTA mice significantly reduced f_R_ ∼40–50% from baseline (*p* = 0.026 and *p* = 0.024, respectively; Holm-Sidak *post hoc*), before increasing towards baseline levels. In striking contrast, aged rTg4510 mice did not change f_R_ during hyperoxia (*p* = 0.950 vs*.* baseline). Once aged nTg and tTA mice aroused ∼20 s into the hyperoxia exposure, they remained awake with active sniffing; thus, f_R_ increased >100% of baseline during quiet rest (both *p* < 0.001), an effect we attribute to arousal versus the direct physiological response to hyperoxia. Interestingly, aged rTg4510 mice did not arouse in response to hyperoxia.

Though a 2-way RM ANOVA revealed no significant interaction between genotype and time in hyperoxia for V_T_, genotype (F_2,22_ = 4.157, *p* = 0.025) and time under hyperoxia (F_2,44_ = 4.919, *p* = 0.010) each had independent effects (Figure 5C). Immediately following hyperoxia onset, aged nTg and tTA mice significantly reduced V_T_ from baseline (*p* = 0.036 and *p* = 0.021, respectively; Holm-Sidak *post hoc*), before returning towards baseline. Aged rTg4510 mice did not change V_T_ during hyperoxia (*p* = 0.943 vs*.* baseline). V_T_ was not significantly altered by arousal vs*.* baseline in aged nTg (*p* = 0.804) or tTA mice (*p* = 0.686).

$\dot{V}$ _E_ was immediately reduced in hyperoxia in aged nTg and tTA, but not rTg4510 mice; there was a significant interaction between genotype and time under hyperoxia for $\dot{V}$ _E_ (F_4,74_ = 4.058, *p* = 0.007; 2-way RM ANOVA; Figure 5D). Aged nTg and tTA mice significantly reduced $\dot{V}$ _E_ from ambient conditions (*p* = 0.028 and *p* = 0.009, respectively; Holm-Sidak *post hoc*), before increasing towards baseline. However, aged rTg4510 mice did not change $\dot{V}$ _E_ during hyperoxia (*p* = 0.829 versus baseline). Similar to f_R_, $\dot{V}$ _E_ significantly increased on arousal in aged nTg and tTA mice (both *p* < 0.001); aged rTg4510 mice did not.

Genotype had significant effects on time to arousal from presumptive sleep after hyperoxia onset (F_2,30_ = 6.029, *p* = 0.006; 1-way ANOVA; Figure 5E); aged rTg4510 mice remained in presumptive sleep for significantly longer periods after the onset of hyperoxia (56.2 ± 13.1 s) vs*.* nTg mice (17.6 ± 2.6 s; *p* = 0.009). Time to arousal of tTA mice was similar to nTg mice (13.9 ± 2.6 s; *p* = 0.802).

### 3.5 Abolished CO_2_ chemosensitivity in aged rTg4510 mice

**FIGURE 6:**
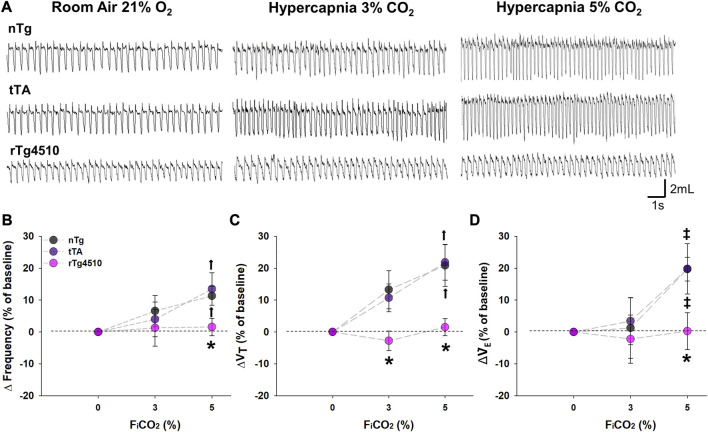
CO_2_ chemosensitivity is absent in aged rTg4510 mice during 3% and 5% CO_2_ hypercapnic challenge. In **(A)**, representative plethysmograph traces of stable breathing during presumptive sleep to room air (21% O_2_; left column), hypercapnia with 3% (middle column) and 5% (right column) CO_2_ hypercapnic exposures in aged nTg (top), tTA (middle), and rTg4510 (bottom) mice. Aged rTg4510 mice did not change their respiratory frequency **(B)**, tidal volume (V_T_), **(C)**, or minute ventilation ( $\dot{V}$ _E_), **(D)** in response to hypercapnia with 3 or 5% CO_2_. *, *p* < 0.050 vs*.* age-matched nTg control; ‡, *p* < 0.050 vs*.* baseline; ‡, *p* < 0.050 vs*.* baseline and 3% CO_2_ challenge. Data presented as mean ± SEM.

Mice were exposed to hypercapnia (3% and 5% CO_2_) for 5 min to test CO_2_ chemosensitivity, an effect normally dominated by central CO_2_ chemoreceptors (Figure 6A). While respiratory frequency was significantly increased from baseline during 5% CO_2_ exposure for aged nTg (11.3% ± 3.0%; *p* = 0.007) and tTa mice (13.5% ± 5.1%; *p* = 0.003; Figure 6B), aged rTg4510 mice showed no significant elevation in f_R_ (1.6% ± 2.7%, *p* = 0.648). Aged rTg4510 mice also show no significant impact of inspired CO_2_ on either V_T_ (*3% CO*_*2*_: 2.8% ± 3.0%, *p* = 0.497; *5% CO*_*2*_: 1.5% ± 2.7%, *p* = 0.744; Figure 6C) or $\dot{V}$ _E_ (*3%* CO_*2*_: 2.2% ± 7.6%, *p* = 0.936; *5%* CO_*2*_: 0.3% ± 5.8%, *p* = 0.971; Figure 6D) versus baseline; in contrast, aged nTg and tTA mice both significantly increased V_T_ (20.9% ± 6.6%; *p* = 0.017 and 21.8% ± 5.6%; *p* = 0.002) and $\dot{V}$ _E_ (19.7% ± 3.7%; *p* = 0.037 and 19.8% ± 7.9%; *p* = 0.036) during 5% inspired CO_2_. There were no individually significant effects of 3% CO_2_ in aged nTg, tTA or rTg4510 mice on f_R_ (6.6% ± 4.8%; *p* = 0.110; 4.0% ± 5.5%; *p* = 0.330; and 1.3% ± 5.7%; *p* = 0.621, respectively), V_T_ (13.2% ± 6.0%; *p* = 0.051; 10.7% ± 4.4%; *p* = 0.131; and −2.8% ± 3.1%; *p* = 0.497, respectively) or $\dot{V}$ _E_ (1.2% ± 9.4%; *p* = 0.825; 3.4% ± 7.4%; *p* = 0.676; and −2.2% ± 7.6%; *p* = 0.936, respectively) versus baseline.

### 3.6 Airway mechanics and lung function

**FIGURE 7:**
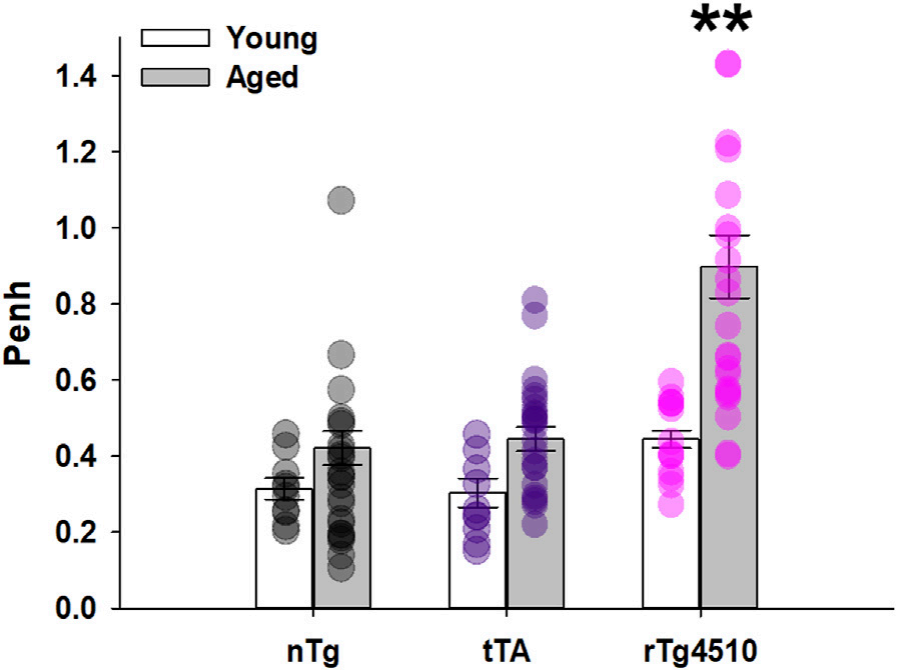
Penh was significantly elevated in aged rTg4510 mice. Relative bronchoconstriction, represented as Penh (enhanced pause) was significantly greater in aged rTg4510 mice vs*.* young rTg4510 mice and age-matched controls (all *p* < 0.001). **, *p* < 0.050 vs*.* all comparisons. Data presented as mean ± SEM.

Penh (enhanced pause) is an arbitrary, dimensionless measure calculated as Pause*(Post-Expiratory Pause/Post-Inspiratory Pause); Penh is suggested to be an indicator of bronchoconstriction (SCIREQ Scientific Respiratory Equipment Inc., Montréal, Canada). Increased bronchoconstriction is considered to be paralleled with an increase in Penh. During presumptive sleep in ambient conditions (21% O_2_), there was a significant interaction between genotype and age on Penh (F_2,103_ = 5.603, *p* = 0.005; 2-way mixed effects ANOVA; Figure 7), driven largely by aged rTg4510 mice. Penh increased in rTg4510 mice from young (0.44 ± 0.02) to aged mice (0.90 ± 0.08; *p* < 0.001); Penh in aged rTg4510 mice was ∼2 fold greater vs*.* aged nTg mice (0.42 ± 0.04; *p* < 0.001), suggesting the possibility of greater airway resistance in aged rTg4510 mice.

**FIGURE 8:**
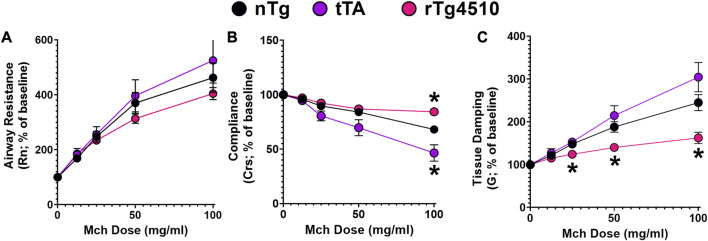
Airway resistance in aged rTg4510 mice does not differ from aged control nTg or tTA mice in response to increasing MCh dose. In **(A)**, airway resistance (Rn) did not differ between aged nTg, tTA and rTg4510 mice. In **(B)**, dynamic compliance (Crs) was only different for aged rTg4510 and tTA mice at the highest methacholine (MCh) dose vs*.* aged nTg controls (both *p* < 0.050). In **(C)**, tissue damping was significantly blunted with increasing MCh doses in aged rTg4510 mice, only, vs*.* aged controls (*p* < 0.035 for all doses). *, *p* < 0.050 vs*.* age-matched nTg control. Data presented as mean ± SEM.

To more directly compare airway mechanics and lung function between aged nTg (n = 10), tTA (n = 10) and rTg4510 (n = 14) mice, *in vivo* respiratory mechanics were assessed using the FlexiVent Forced Oscillation Technique in anesthesized, tracheostomized and ventilated mice as described previously (Reznikov et al., 2016; Reznikov et al., 2018a). Airway resistance, dynamic compliance [indicators of bronchoconstriction (Zhang et al., 2009)] and tissue damping using the Constant Phase Model (Hantos et al., 1992) were assessed to obtain a parametric distinction between differences in airway vs*.* tissue mechanics (Figure 8).

Basal measurements of airway resistance (nTg: 0.27 ± 0.07 cmH_2_O•s•mL^−1^; tTA: 0.26 ± 0.04; rTg4510: 0.20 ± 0.02; F_2,31_ = 0.873, *p* = 0.428; 1-way ANOVA), dynamic compliance (nTg: 0.07 ± 0.003 mL•cmH_2_O^−1^; tTA: 0.08 ± 0.002; rTg4510: 0.07 ± 0.003; F_2,31_ = 1.163, *p* = 0.326) and tissue damping (nTg: 2.88 ± 0.12; tTA: 2.85 ± 0.24; rTg4510: 3.02 ± 0.10; F_2,31_ = 0.341, *p* = 0.714) were similar between all groups. While there was a dose-dependent methacholine effect on airway resistance (F_1,42_ = 119.8, *p* < 0.001), genotype had no effect on methacholine response magnitude (F_2,31_ = 1.097, *p* = 0.347; Figure 8A).

Dynamic compliance describes the ease with which the respiratory system expands/contracts and characterizes elastic properties that must be overcome to breathe (Lum and Mitzner, 1987). There was a significant interaction between genotype and methacholine dose on dynamic compliance (F_8,122_ = 9.885, *p* < 0.001; Figure 8B); at the highest methacholine dose (100 mg/mL), aged rTg4510 had less reduction in dynamic lung compliance (84.3% ± 4.8%) versus nTg mice (68.1% ± 8.2%; *p* = 0.005). In aged tTA mice, dynamic lung compliance (46.4% ± 4.3%) was reduced more than in nTg mice (*p* = 0.042).

Tissue damping is related to tissue resistance and reflects the energy dissipation in the alveoli (Hantos et al., 1992; Collins et al., 2003). There was a significant interaction between genotype and methacholine dose on tissue damping (F_8,122_ = 9.649, *p* < 0.001; Figure 8C). Overall, aged rTg4510 mice (versus aged nTg mice) had less gain of tissue damping with increasing methacholine doses: 25 mg/mL (124% ± 4% and 148% ± 9%, *p* = 0.033), 50 mg/mL (140% ± 7% and 188% ± 14%, *p* = 0.008) and 100 mg/mL (162% ± 13% and 245% ± 20%, *p* = 0.004).

**FIGURE 9:**
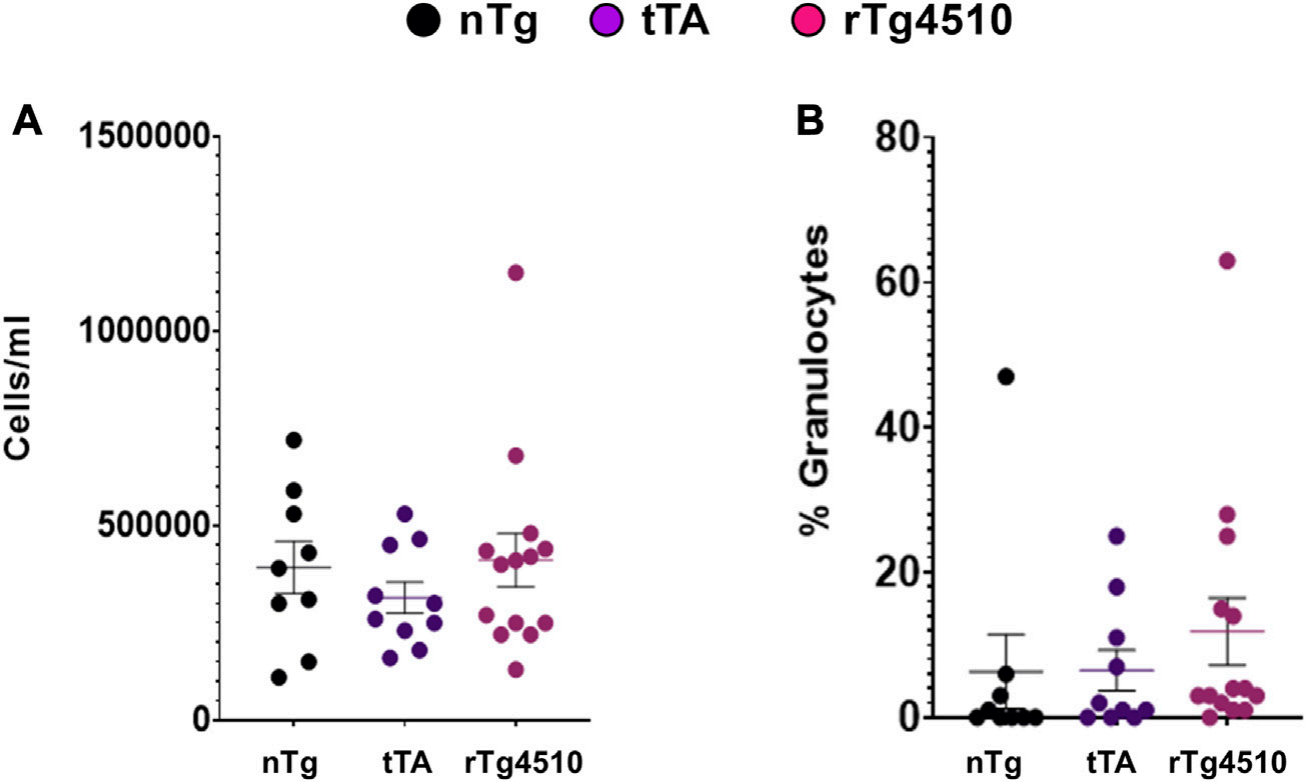
Cells from bronchoalveolar lavage fluid are similar between aged nontransgenic (nTg), monogenic tTA, and rTg4510 mice. Bronchoalveolar lavage fluid was assayed for the total number of cells **(A)** and percentage of granulocyte cells **(B)**. Data presented as mean ± SEM.

To determine if there was potential infiltration of inflammatory mediators contributing to genotype differences in resistance and lung function, bronchoalveolar lavage fluid was assayed for total cell number and percentage of granulocytes at the end of flexiVent experiments (Figure 9; (Reznikov et al., 2018b). There was no difference between genotypes in total number of cells/mL (F_2,30_ = 0.656, *p* = 0.526; 1-way ANOVA; Figure 9A) or percentage of granulocyte cells (F_2,30_ = 0.551, *p* = 0.582; Figure 9B). Follow-up histological analyses of airway structure to assess the mean free distance between gas exchange surfaces (*i.e.,* alveoli structure/complexity) indicate no major difference in lung structure between genotypes (data not shown).

### 3.7 Hyperphosphorylated tau in the brainstem of aged rTg4510 mice

**FIGURE 10:**
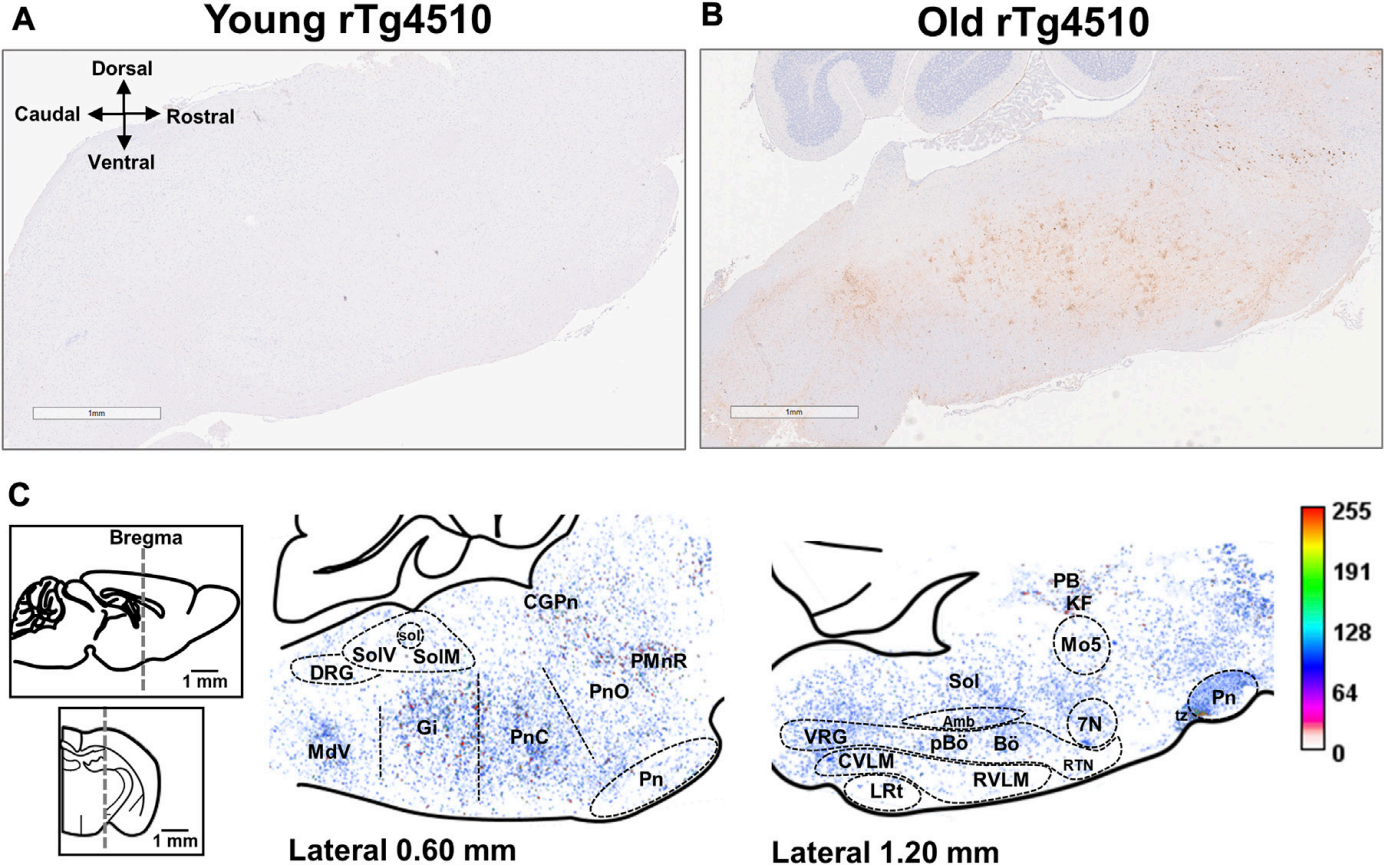
Aged rTg4510 mice have advanced tau pathology in the brainstem and midbrain regions involved in respiratory rhythm generation and chemoreflex function. Representative images for AT-100 staining (hyperphosphorylated tau) in young **(A)** and aged **(B)** rTg4510 mice (n = 3 each group; scale bar is 1 mm). In **(C)**, are heat maps containing average AT-100 staining from aged rTg4510 mice in the brainstem and midbrain, overlaid onto the reference brain section image (Paxinos, 2001) 0.60 mm (left image) and 1.20 mm (right image) lateral to midline, to estimate the distribution of hyperphosphorylated tau. In the corresponding intensity scale, dark red indicates maximal AT-100, and light beige/pink indicates minimal AT-100. Abbreviations: facial motor nucleus (7N); nucleus ambiguus (Amb); Bötzinger complex (Bö); central gray substance of the pons (CPgN); caudal ventrolateral medulla oblongata (CVLM); dorsal respiratory group (DRG); gigantocellular reticular nucleus (Gi); Kölliker-Fuse nucleui (KF); lateral reticular nucleus (LRt); trigeminal motor nucleus (Mo5); parabrachial complex (PB); pre-Bötzinger complex (pBö); raphe magnus of the pons (PMnR); oral and caudal parts of the pontine reticular nucleus (PnO; PnC); retrotrapezoid nucleus (RTN); rostral ventrolateral medulla oblongata (RVLM); nucleus of the solitary tract (Sol); medial nucleus of tractus solitarius (SolM); ventrolateral nucleus of the tractus solitarius (SolV); ventral respiratory group (VRG).

Staining for hyperphosphorylated tau of [AT-100 (pT212/S214)] was done in young and old rTg4510 mice (both n = 3) to verify tau pathology in regions of the brainstem respiratory circuit (Figure 10). We did not observe AT-100 staining in the brainstems of young rTg4510 mice (Figure 10A). However, hyperphosphorylated tau was observed in brainstem regions associated with chemosensory function (*e.g.,* retrotrapezoid nucleus) and respiratory pattern/rhythm generation (*e.g.*, pre-Bötzinger Complex) in aged rTg4510 mice (Figure 10B). High staining density was also observed in pontine, dorsal and ventral respiratory groups (Figure 10C).

#### 3.7.1 Ponto-medullary reticular formation

Tauopathy was robust in the dorsal and caudal parts of the pontine reticular nucleus. Heavily stained regions included the Kölliker-Fuse nucleus of the parabrachial complex and gigantocellular reticular nucleus, though staining was sparse in the lateral reticular nucleus. Tauopathy was also evident in the dorsal respiratory group (*i.e*., ventrolateral nucleus of the tractus solitarius) and the ventral respiratory column near the nucleus ambiguous, which includes the rostral and caudal ventral respiratory groups, the retrotrapezoid nucleus, the pre-Bötzinger and the Bötzinger complexes (Alheid and McCrimmon, 2008).

Tauopathy-laden neurons were also evident in the: 1) ponto-medullary reticular arousal system, including raphe magnus and central gray of the pons; 2) cranial motor nuclei, including nucleus ambiguus, trigeminal motor nucleus and facial motor nucleus; and 3) medial and ventrolateral parts of the nucleus of the solitary tract. In contrast, the cerebellum was devoid of tau pathology in both young and aged rTg4510 mice, as previously described (Ramsden et al., 2005).

## 4 Discussion

Our primary goal was to test the hypothesis that development and progression of tauopathy causes dysfunction in the neural system controlling breathing. We noted a profound increase in breathing irregularity (increased sighs and apneas) and near total loss of peripheral and central chemoreceptor function during presumptive sleep. Although these profound changes were not associated with overt neurodegeneration in brainstem regions known to be associated with respiratory rhythm generation, pattern formation or chemoreflexes, they were clearly associated with age-related accumulation of hyperphosphorylated tau in these key regions of aged mice, suggesting the potential for pathophysiology that had not yet progressed to overt neurodegeneration. We are not aware of similar deficits in other murine models of tauopathy (and by inference, Alzheimer’s disease).

We demonstrate that irregular breathing and loss of chemoreflexes cannot be explained by impaired lung/airway mechanics or inflammation. Thus, the extension of age-dependent tauopathy to brainstem regions controlling breathing (including areas that mediate respiratory chemoreflexes) likely gives rise to the respiratory phenotype of rTg4510 mice. We conclude age-dependent brainstem tauopathy increases breathing instability, including increased incidence of sighs, apneas and hypopneas, and profoundly suppresses peripheral and central chemoreflexes. Similar effects may contribute to the unstable breathing typically observed during sleep in humans with tauopathy, including those afflicted with Alzheimer’s Disease.

### 4.1 Progressive tauopathy disrupts breathing pattern and impairs chemoreflex function

#### 4.1.1 Peripheral chemoreflex function

Changes in PO_2_ are sensed rapidly by peripheral arterial chemoreceptors, particularly carotid body chemoreceptors located near the carotid bifurcation (Kumar and Prabhakar, 2012). These chemoreceptors are dominant contributors to hypoxic chemoreflexes, although they also respond rapidly to changes in arterial PCO_2_. Heightened carotid body chemoreceptor activity is often associated with sleep disordered breathing (Orr et al., 2017; Peng et al., 2017). Whereas sleep apnea most often arises from chemoreflex hypersensitivity [*i.e.,* high loop gain (Orr et al., 2017)], progressive tauopathy in aged rTg4510 mice virtually abolished peripheral and central chemoreflexes, effectively abolishing loop gain. With brainstem tauopathy in this murine model system, the “watchdogs” are sleeping, leaving aged rTg4510 mice unable to respond to perturbations in these powerful breathing stimuli (*i.e.*, O_2_ and CO_2_).

Hyperoxia suppresses peripheral chemoreceptor activity, transiently reducing the drive to breathe (*i.e.,* the “Dejours” test; *see* (Skatrud and Dempsey, 1983; Bouferrache et al., 2000). In normal humans, decreased ventilation occurs immediately on 100% O_2_ administration, and secondarily increases arterial PCO_2_, which partially restores ventilation towards baseline levels (Lahiri et al., 1978; Skatrud and Dempsey, 1983; Edwards et al., 2014). As expected, aged nTg and tTA control mice both displayed rapid reductions in ventilation on breathing 100% oxygen, with a gradual increase towards baseline levels similar to humans. In contrast, old rTg4510 mice had virtually no response to 100% inspired oxygen, demonstrating complete abolition of peripheral chemoreflex function.

In humans with high “loop gain”, chemoreflex activation during apneic episodes causes arousal, providing an additional drive to breathe (the “wakefulness drive”); thus, arousal ends the apnea and causes a ventilatory overshoot that perpetuates the cycle of apnea-hyperventilation-apnea (Phillipson, 1978; Skatrud and Dempsey, 1983; Horner et al., 2001; Orr et al., 2017). Something quite distinct was apparent in rTg4510 mice. Both nTg and tTA control mice exhibited hypopnea within ∼20 s of breathing 100% oxygen, and then aroused for unknown reasons. Once aroused, control mice remained awake until the hyperoxic exposure ended. In striking contrast, old rTg4510 mice exhibited neither hypoventilation nor arousal when breathing 100% oxygen. Although it is not clear what the “wake up” call may be during 100% oxygen breathing, we suggest that hypercapnia developing during the O_2_-induced hypopnea may trigger arousal since: 1) carotid bodies are suppressed, not stimulated by hyperoxia; and 2) failure to arouse in rTg4510 mice is consistent with their lack of hypercapnic chemoreflex function. On the other hand, arousal in control mice may have been due to the mechanical sensation of reduced ventilation *per se* (Horner et al., 2001; Trinder et al., 2006; Edwards et al., 2014), or by changes in gas odor or humidity when changing to 100% inspired O_2_. Old rTg4510 mice may have lost their ability to detect hypoventilation and/or odor, diminishing the trigger for arousal. Consistent with this idea, tauopathy infiltrates the olfactory bulb early in rTg4510 mice (Kim et al., 2017) and, thus, may interfere with odor detection.

#### 4.1.2 Hypercapnic chemoreflexes

The predominant CO_2_ chemo-sensors are located in the central nervous system (*i.e*., central chemoreceptors), particularly in the medulla (*e.g.*, retrotrapezoid nucleus of ventrolateral medulla (Guyenet et al., 2012) or medullary raphe neurons (Hodges and Richerson, 2008) and dictate resting breathing under normal circumstances (Orr et al., 2017). Hypercapnia activates central chemoreceptors, which dominate the CO_2_ chemoreflex (Bouverot et al., 1963). When using varied levels of inspired CO_2_ to test CO_2_/central chemosensitivity, old nTg and tTA mice increased ventilation from baseline during CO_2_ inhalation as expected. In striking contrast, the lack of response to hypercapnic exposures suggests that key brainstem nuclei essential for CO_2_ ventilatory chemoreflexes are dysfunctional in old rTg4510 mice. It remains to be explored if this lack of chemosensitivity can account for breathing instability in rTg4510 mice during presumptive sleep.

With an apparent elimination of peripheral and central chemoreflexes in the aged rTg4510 mice, total breathing cessation during sleep may be expected, as observed in individuals with congenital central hypoventilation syndrome (CCHS). Unlike CCHS however where there is genetic (developmental) mutation/deactivation of the *PHOX2B* gene, we suspect in this tauopathy model, loss of chemoreflex function occurs late in life. Thus, breathing is likely maintained in the rTg4510 mice by compensatory mechanisms that may have been able to prevent cessation of breathing during sleep. This remains to be explored.

### 4.2 Tauopathy in rTg4510 mice

There are two mainstream hypotheses for the pathogenesis of Alzheimer’s Disease: 1) the amyloid-β hypothesis posits amyloid-β accumulation initiates and drives tau pathology, causing neurodegeneration (Hardy and Selkoe, 2002); versus 2) the tau hypothesis postulates tau phosphorylation and aggregation initiates neurodegeneration (Arnsten et al., 2021). Neurodegenerative diseases such as Alzheimer’s and other dementias are closely linked with tau hyperphosphorylation and *postmortem* findings of neurofibrillary tangles (Braak and Braak, 1991; 1995; Dutschmann et al., 2010; Braak et al., 2011; Braak and Del Tredici, 2015). Although connections between tau phosphorylation or tauopathy and clinical symptoms are well understood in pathological detail (Terwel et al., 2002), pathophysiological findings (distinct from neurodegeneration) have seldom been considered.

In many primary and secondary tauopathies, there are substantial regional differences in affected CNS structures. As yet, neither human patient nor animal model investigations have yielded definitive explanations for differential brain circuit susceptibility to tau pathology. *Postmortem* analysis of brains from humans with tauopathy demonstrate tau aggregation in widespread regions, including the brainstem (Eser et al., 2018). Here, we analyzed a transgenic mouse strain with neuron-specific, postnatal over-expression of mutant human Tau-P301L (Ramsden et al., 2005; Santacruz et al., 2005), rTg4510 mice. In old rTg4510 mice, a consistent pattern of tau protein phospho-epitope AT-100 was observed within defined nuclei of the pons and medulla. Many of these same structures are closely linked to the regulation of breathing and airway function (Dutschmann et al., 2010; Eser et al., 2018), including regions associated with respiratory rhythm generation and chemoreflex function. Thus, although we did not observe neurodegeneration in these regions *per se*, accumulation of hyperphosphorylated tau protein suggest a basis for the profound dysfunction in the neural control of breathing reported here.

Tauopathy was detected in the pontine Kolliker-Fuse nucleus, which plays an essential role in the regulation of post-inspiratory motor activity (Dutschmann et al., 2008) and could contribute to respiratory dysrhythmia and intermittent apneas (Lalley, 2007). Tau pathology was also observed in primary medullary respiratory centers, including the ventral respiratory column, which includes the primary rhythm generating area known as the preBötzinger complex (Smith et al., 1991; Feldman et al., 2003). Serotonergic medullary raphe nuclei have been implicated in arousal, central chemosensitivity and state-dependent modulation of breathing (Hodges et al., 2008; Hodges and Richerson, 2008). The retrotrapezoid nucleus is a critical element of the CNS network in central respiratory chemoreflexes that depend on serotonin input (Mulkey et al., 2007; Guyenet et al., 2012). Tauopathy in any of these key regions may modify/interfere with normal functions of rhythm generation and chemoreception, manifesting as frequent sighs, post-sigh and spontaneous apneas, and the near abolition of peripheral and central chemoreflexes.

Though AT-100 staining in the brainstem of aged rTg4510 mice is not as robust as the hippocampus and cortex [not shown; *see* (Ramsden et al., 2005; Santacruz et al., 2005)], there is sufficient hyperphosphorylated tau and pre-tangle pathology to suspect pathophysiology. Even without visible brainstem tau pathology in young rTg4510 mice using our methods, increased apnea frequency and abolition of chemoreflexes suggest that brainstem pathophysiology precedes visible neurodegeneration in rTg4510 mice, which could align with the concept of early toxic species, such as “seeds” or oligomers.

Recent reports indicate gene disruption with transgene insertion of both the TAU_P301L_-TgINDEL (1 disruption) and tTA-TgINDEL (5 disruptions) mutations in mouse, and suggest that endogenous gene disruption contributes to the pathophysiological phenotype of rTg4510 mice (Gamache et al., 2019; Barabas et al., 2022). Since the tTA-TgINDEL allele alone is sufficient to drive progressive neuron loss with obvious degeneration in the dentate gyrus (Han et al., 2012), we included tTA mice as a secondary control and found no difference in results versus nTg mice. While we cannot completely rule out the possibility that endogenous gene disruption in rTg4510 could account for some observed effects, our findings demonstrate age-dependent tauopathy and breathing deficits that are unique to the rTg4510 genotype.

### 4.3 Lung and airway dysfunction in aged rTg4510 mice

Measurements of lung/airway function indicate that airway resistance in aged rTg4510 mice cannot account for pathophysiological breathing responses reported here. However, aged rTg4510 mice do have greater compliance and reduced tissue damping versus age-matched control mice. Although it is difficult to link changes in lung compliance or tissue damping with loss of chemoreflexes reported here, it remains possible that changes in lung mechanics alter sensory feedback from the lungs, contributing to the incidence of sighs and/or apneas. Further investigations of rTg4510 mice with pulmonary denervation would be helpful in ruling out this unlikely possibility. Although there was a lack of major differences in lung structure or mechanics between genotypes, some differences in autonomic nervous system engagement became apparent with methacholine administration. Thus, we cannot rule out the possibility of subtle lung structural differences that only become apparent when the system is challenged or stressed.

The rTg4510 mice develop a hunched posture with aging and the progression of tauopathy by 9 months of age (Ramsden et al., 2005; Santacruz et al., 2005), consistent with observations in aged humans with advanced dementia (Galasko et al., 1990; Leibovitz et al., 1993). Although lung mechanics could not explain the hunched posture of old rTg4510 mice, that postural change could interfere with chest wall mechanics and, therefore, lung expansion/contraction. This possibility remains to be explored.

### 4.4 Limitations

Ventilation can be influenced by intrinsic (*e.g.*, body weight, body temperature, metabolic rate) (Piccione et al., 2005; Liu et al., 2009) and extrinsic factors (*e.g.,* ambient temperature, altitude) (Bouverot, 1985; Mortola, 2005). In the present studies we normalized ventilation and tidal volume to body mass, and accounted for differences in body temperature, level of physical activity (by analyzing breaths in quiet rest/presumptive sleep) as well as ambient temperature/humidity and barometric pressure. All measurements were made during the day, the rest phase of mice. Standardizing time of day is important since circadian rhythm and/or sleep influences breathing (Mortola and Seifert, 2002; Mortola, 2007; Adamczyk et al., 2008). One limitation of this study is that we did not measure O_2_ consumption or CO_2_ production during baseline or chemoreflex challenges. Mice have high metabolic rates per Gram of tissue, and metabolic rate declines in a number of conditions, including hypoxia (Mortola, 1993; Speakman, 2013; Gu and June 2018). Thus, changes in ventilation during hypoxia are impacted by hypometabolism [“hypoxic hypometabolism;” (Mortola, 1993; Speakman, 2013; Gu and June 2018)] and associated hypocapnia, obscuring the peripheral ventilatory chemoreflex. To avoid this complication, we used transient hyperoxia (the “Dejours test”) to assess peripheral chemoreflex responses. However, future investigation should take consider metabolic rate.

Although video aided us in determining “presumptive sleep”, assessment of sleep disturbances during different sleep states using EEG may provide more information in the future. For example, “EEG slowing” during wake and REM sleep and a degradation of non-REM EEG microstructure have been reported in people with Alzheimer’s Diseases and obstructive sleep apnea (Mullins et al., 2020). Generally, obstructive sleep apnea tends to be more severe during REM (vs. non-REM) sleep (Mahmood et al., 2009), but central apneas are less frequent during REM (vs. non-REM) sleep (Ludwig et al., 2023).

Mice do not inherently have obstructive sleep apnea but can exhibit central apneas at rates that depend on sleep state, genetics, and strain (Davis and O'Donnell, 2013; Kim et al., 2019). Thus, using EEG in our studies would have aided in assignment of apneas to sleep states. Nonetheless, it is clear breathing disturbances in the aged rTg4510 mice are prominent compared to young, and age-matched controls.

## 5 Conclusion

We demonstrate profound breathing instability (sighs and apneas) and impaired chemoreflex function in aged rTg4510 mice with age-related accumulation of hyperphosphorylated tau in defined neural circuits controlling respiratory pattern and chemosensitivity. We suggest that once hyperphosphorylated tau infiltrates brainstem regions controlling respiratory rhythm, pathophysiology ensues (prior to neurodegeneration), increasing the incidence of sighs, apneas and hypopneas. Penetration to other key areas likely explains profound suppression of peripheral and central chemoreflexes. Similar effects may contribute to unstable breathing characteristic of human tauopathy patients during sleep. In turn, breathing dysregulation may exacerbate tauopathy progression.

## Data Availability

The original contributions presented in the study are included in the article, further inquiries can be directed to the corresponding author.

## References

[B1] Adamczyk W. Tafil-Klawe M. Siekierka M. Zlomanczuk P. Weber P. Klawe J. J. (2008). Daily pattern of breathing in healthy young men. J. Physiol. Pharmacol. 59 (6), 115–122. 19218635

[B2] Albdewi M. A. Liistro G. El Tahry R. (2018). Sleep-disordered breathing in patients with neuromuscular disease. Sleep. Breath. 22 (2), 277–286. 10.1007/s11325-017-1538-x 28702830

[B3] Alheid G. F. McCrimmon D. R. (2008). The chemical neuroanatomy of breathing. Respir. Physiol. Neurobiol. 164 (1-2), 3–11. 10.1016/j.resp.2008.07.014 18706532 PMC2701569

[B4] Andrade A. G. Bubu O. M. Varga A. W. Osorio R. S. (2018). The relationship between obstructive sleep apnea and alzheimer's disease. J. Alzheimers Dis. 64 (1), S255–S270. 10.3233/JAD-179936 29782319 PMC6542637

[B5] Arnsten A. F. T. Datta D. Del Tredici K. Braak H. (2021). Hypothesis: tau pathology is an initiating factor in sporadic alzheimer's disease. Alzheimers Dement. 17 (1), 115–124. 10.1002/alz.12192 33075193 PMC7983919

[B6] Bahia C. Pereira J. S. (2015). Obstructive sleep apnea and neurodegenerative diseases: A bidirectional relation. Dement. Neuropsychol. 9 (1), 9–15. 10.1590/S1980-57642015DN91000003 29213936 PMC5618986

[B7] Barabas A. J. Robbins L. A. Gaskill B. N. (2022). Home cage measures of Alzheimer's disease in the rTg4510 mouse model. Genes Brain Behav. 21 (2), e12795. 10.1111/gbb.12795 35044727 PMC9744509

[B8] Bliwise D. L. (2004). Sleep disorders in Alzheimer's disease and other dementias. Clin. Cornerstone 6 (1A), S16–S28. 10.1016/s1098-3597(04)90014-2 15259536

[B9] Bouferrache B. Filtchev S. Leke A. Marbaix-Li Q. Freville M. Gaultier C. (2000). The hyperoxic test in infants reinvestigated. Am. J. Respir. Crit. Care Med. 161 (1), 160–165. 10.1164/ajrccm.161.1.9904012 10619814

[B10] Bouverot P. (1985). Adaptation to altitude-hypoxia in vertebrates. Berlin, Germany: Springer-Verlag. 10.1007/978-3-642-82316-9

[B11] Bouverot P. Puccinelli R. Flandrois R. Papiernik E. (1963). Mechanisms of action of CO-2 stimulation of respiration during prolonged hypercapnia. J. Physiol. Paris. 55, 207–208. 14014548

[B12] Braak H. Braak E. (1991). Neuropathological stageing of Alzheimer-related changes. Acta Neuropathol. 82 (4), 239–259. 10.1007/BF00308809 1759558

[B13] Braak H. Braak E. (1995). Staging of Alzheimer's disease-related neurofibrillary changes. Neurobiol. Aging 16 (3), 271–278. 10.1016/0197-4580(95)00021-6 7566337

[B14] Braak H. Del Tredici K. (2015). Neuroanatomy and pathology of sporadic Alzheimer's disease. Adv. Anat. Embryol. Cell Biol. 215, 1–162. 10.1007/978-3-319-12679-1 25920101

[B15] Braak H. Thal D. R. Ghebremedhin E. Del Tredici K. (2011). Stages of the pathologic process in alzheimer disease: age categories from 1 to 100 years. J. Neuropathol. Exp. Neurol. 70 (11), 960–969. 10.1097/NEN.0b013e318232a379 22002422

[B16] Carvalho D. Z. St Louis E. K. Schwarz C. G. Lowe V. J. Boeve B. F. Przybelski S. A. (2020). Witnessed apneas are associated with elevated tau-PET levels in cognitively unimpaired elderly. Neurology 94 (17), e1793–e1802. 10.1212/WNL.0000000000009315 32217775 PMC7274847

[B17] Collins R. A. Ikegami M. Korfhagen T. R. Whitsett J. A. Sly P. D. (2003). *In vivo* measurements of changes in respiratory mechanics with age in mice deficient in surfactant protein D. Pediatr. Res. 53 (3), 463–467. 10.1203/01.PDR.0000049464.46191.BF 12595595

[B18] Daulatzai M. A. (2015). Evidence of neurodegeneration in obstructive sleep apnea: relationship between obstructive sleep apnea and cognitive dysfunction in the elderly. J. Neurosci. Res. 93 (12), 1778–1794. 10.1002/jnr.23634 26301370

[B19] Davis E. M. O'Donnell C. P. (2013). Rodent models of sleep apnea. Respir. Physiol. Neurobiol. 188 (3), 355–361. 10.1016/j.resp.2013.05.022 23722067 PMC4010146

[B20] Dempsey J. A. Veasey S. C. Morgan B. J. O'Donnell C. P. (2010). Pathophysiology of sleep apnea. Physiol. Rev. 90 (1), 47–112. 10.1152/physrev.00043.2008 20086074 PMC3970937

[B21] Dutschmann M. Menuet C. Stettner G. M. Gestreau C. Borghgraef P. Devijver H. (2010). Upper airway dysfunction of Tau-P301L mice correlates with tauopathy in midbrain and ponto-medullary brainstem nuclei. J. Neurosci. 30 (5), 1810–1821. 10.1523/JNEUROSCI.5261-09.2010 20130190 PMC6633985

[B22] Dutschmann M. Morschel M. Reuter J. Zhang W. Gestreau C. Stettner G. M. (2008). Postnatal emergence of synaptic plasticity associated with dynamic adaptation of the respiratory motor pattern. Respir. Physiol. Neurobiol. 164 (1-2), 72–79. 10.1016/j.resp.2008.06.013 18620081

[B23] Edwards B. A. Sands S. A. Owens R. L. White D. P. Genta P. R. Butler J. P. (2014). Effects of hyperoxia and hypoxia on the physiological traits responsible for obstructive sleep apnoea. J. Physiol. 592 (20), 4523–4535. 10.1113/jphysiol.2014.277210 25085887 PMC4287742

[B24] Eser R. A. Ehrenberg A. J. Petersen C. Dunlop S. Mejia M. B. Suemoto C. K. (2018). Selective vulnerability of brainstem nuclei in distinct tauopathies: A postmortem study. J. Neuropathol. Exp. Neurol. 77 (2), 149–161. 10.1093/jnen/nlx113 29304218 PMC6251636

[B25] Feldman J. L. Mitchell G. S. Nattie E. E. (2003). Breathing: rhythmicity, plasticity, chemosensitivity. Annu. Rev. Neurosci. 26, 239–266. 10.1146/annurev.neuro.26.041002.131103 12598679 PMC2811316

[B26] Galasko D. Kwo-on-Yuen P. F. Klauber M. R. Thal L. J. (1990). Neurological findings in Alzheimer's disease and normal aging. Arch. Neurol. 47 (6), 625–627. 10.1001/archneur.1990.00530060033012 2346387

[B27] Gamache J. Benzow K. Forster C. Kemper L. Hlynialuk C. Furrow E. (2019). Factors other than hTau overexpression that contribute to tauopathy-like phenotype in rTg4510 mice. Nat. Commun. 10 (1), 2479. 10.1038/s41467-019-10428-1 31171783 PMC6554306

[B28] Gossen M. Bujard H. (1992). Tight control of gene expression in mammalian cells by tetracycline-responsive promoters. Proc. Natl. Acad. Sci. U. S. A. 89 (12), 5547–5551. 10.1073/pnas.89.12.5547 1319065 PMC49329

[B29] Gu C. Jun J. C. (2018). Does hypoxia decrease the metabolic rate? Front. Endocrinol. (Lausanne) 9, 668. 10.3389/fendo.2018.00668 30555410 PMC6282065

[B30] Guyenet P. G. Stornetta R. L. Abbott S. B. Depuy S. D. Kanbar R. (2012). The retrotrapezoid nucleus and breathing. Adv. Exp. Med. Biol. 758, 115–122. 10.1007/978-94-007-4584-1_16 23080151 PMC5111164

[B31] Han H. J. Allen C. C. Buchovecky C. M. Yetman M. J. Born H. A. Marin M. A. (2012). Strain background influences neurotoxicity and behavioral abnormalities in mice expressing the tetracycline transactivator. J. Neurosci. 32 (31), 10574–10586. 10.1523/JNEUROSCI.0893-12.2012 22855807 PMC3431916

[B32] Hantos Z. Daroczy B. Suki B. Nagy S. Fredberg J. J. (1992). Input impedance and peripheral inhomogeneity of dog lungs. J. Appl. Physiol. 72(1), 168–178. 10.1152/jappl.1992.72.1.168 1537711

[B33] Hardy J. Selkoe D. J. (2002). The amyloid hypothesis of alzheimer's disease: progress and problems on the road to therapeutics. Science 297 (5580), 353–356. 10.1126/science.1072994 12130773

[B34] Hodges M. R. Richerson G. B. (2008). Contributions of 5-HT neurons to respiratory control: neuromodulatory and trophic effects. Respir. Physiol. Neurobiol. 164 (1-2), 222–232. 10.1016/j.resp.2008.05.014 18595785 PMC2642893

[B35] Hodges M. R. Tattersall G. J. Harris M. B. McEvoy S. D. Richerson D. N. Deneris E. S. (2008). Defects in breathing and thermoregulation in mice with near-complete absence of central serotonin neurons. J. Neurosci. 28 (10), 2495–2505. 10.1523/JNEUROSCI.4729-07.2008 18322094 PMC6671195

[B36] Horner R. L. Rivera M. P. Kozar L. F. Phillipson E. A. (2001). The ventilatory response to arousal from sleep is not fully explained by differences in CO(2) levels between sleep and wakefulness. J. Physiol. 534 (3), 881–890. 10.1111/j.1469-7793.2001.00881.x 11483717 PMC2278730

[B37] Jacky J. P. (1978). A plethysmograph for long-term measurements of ventilation in unrestrained animals. J. Appl. Physiol. Respir. Environ. Exerc Physiol. 45 (4), 644–647. 10.1152/jappl.1978.45.4.644 101497

[B38] Jensen V. N. Seedle K. Turner S. M. Lorenz J. N. Crone S. A. (2019). V2a neurons constrain extradiaphragmatic respiratory muscle activity at rest. eNeuro 6 (4), 0492–518.2019. 10.1523/ENEURO.0492-18.2019 PMC6709210 31324674

[B39] KamK.JunJ.ParekhA.BubuO. M.MullinsA. E.GuC. (2022). Acute OSA impacts diurnal alzheimer's biomarkers through nocturnal hypoxemia and state transitions. Am. J. Respir. Crit. Care Med. 206 (8), 1039–1042. 10.1164/rccm.202202-0262LE 35696622

[B40] Kazim S. F. Sharma A. Saroja S. R. Seo J. H. Larson C. S. Ramakrishnan A. (2022). Chronic intermittent hypoxia enhances pathological tau seeding, propagation, and accumulation and exacerbates alzheimer-like memory and synaptic plasticity deficits and molecular signatures. Biol. Psychiatry 91 (4), 346–358. 10.1016/j.biopsych.2021.02.973 34130857 PMC8895475

[B41] Kim J. Choi I. Y. Duff K. E. Lee P. (2017). Progressive pathological changes in neurochemical profile of the Hippocampus and early changes in the olfactory bulbs of tau transgenic mice (rTg4510). Neurochem. Res. 42 (6), 1649–1660. 10.1007/s11064-017-2298-5 28523532 PMC5565734

[B42] Kim L. J. Freire C. Fleury Curado T. Jun J. C. Polotsky V. Y. (2019). The role of animal models in developing pharmacotherapy for obstructive sleep apnea. J. Clin. Med. 8 (12), 2049. 10.3390/jcm8122049 31766589 PMC6947279

[B43] Kumar P. Prabhakar N. R. (2012). Peripheral chemoreceptors: function and plasticity of the carotid body. Compr. Physiol. 2 (1), 141–219. 10.1002/cphy.c100069 23728973 PMC3919066

[B44] Lahiri S. Mokashi A. Delaney R. G. Fishman A. P. (1978). Arterial PO2 and PCO2 stimulus threshold for carotid chemoreceptors and breathing. Respir. Physiol. 34 (3), 359–375. 10.1016/0034-5687(78)90134-2 705089

[B45] Lajoie A. C. Lafontaine A. L. Kimoff R. J. Kaminska M. (2020). Obstructive sleep apnea in neurodegenerative disorders: current evidence in support of benefit from sleep apnea treatment. J. Clin. Med. 9 (2), 297. 10.3390/jcm9020297 31973065 PMC7073991

[B46] Lal C. Ayappa I. Ayas N. Beaudin A. E. Hoyos C. Kushida C. A. (2022). The link between obstructive sleep apnea and neurocognitive impairment: an official American thoracic society workshop report. Ann. Am. Thorac. Soc. 19 (8), 1245–1256. 10.1513/AnnalsATS.202205-380ST 35913462 PMC9353960

[B47] Lalley P. M. (2007). Post-inspiratory discharges are the centrepiece of respiratory disrhythmia in a gene knockout model of Rett syndrome. J. Physiol. 579 (3), 565. 10.1113/jphysiol.2007.128249 17234704 PMC2151375

[B48] Lechat B. Naik G. Reynolds A. Aishah A. Scott H. Loffler K. A. (2022). Multinight prevalence, variability, and diagnostic misclassification of obstructive sleep apnea. Am. J. Respir. Crit. Care Med. 205 (5), 563–569. 10.1164/rccm.202107-1761OC 34904935 PMC8906484

[B49] Leibovitz A. Schwartz J. Rosenfeld V. Lerman Y. Habot B. (1993). Acute stooped position in elderly with Alzheimer's disease. J. Am. Geriatr. Soc. 41 (4), 468. 10.1111/j.1532-5415.1993.tb06969.x 8463544

[B50] Liu Q. Fehring C. Lowry T. F. Wong-Riley M. T. (2009). Postnatal development of metabolic rate during normoxia and acute hypoxia in rats: implication for a sensitive period. J. Appl. Physiol. 106(4), 1212–1222. 10.1152/japplphysiol.90949.2008 19118157 PMC2698639

[B51] Ludwig K. Malatantis-Ewert S. Huppertz T. Bahr-Hamm K. Seifen C. Pordzik J. (2023). Central apneic event prevalence in REM and NREM sleep in OSA patients: A retrospective, exploratory study. Biol. (Basel) 12 (2), 298. 10.3390/biology12020298 PMC9953334 36829574

[B52] Lum H. Mitzner W. (1987). A species comparison of alveolar size and surface forces. J. Appl. Physiol. 62(5), 1865–1871. 10.1152/jappl.1987.62.5.1865 3597260

[B53] Lutfi M. F. (2017). The physiological basis and clinical significance of lung volume measurements. Multidiscip. Respir. Med. 12, 3. 10.1186/s40248-017-0084-5 28194273 PMC5299792

[B54] Mahmood K. Akhter N. Eldeirawi K. Onal E. Christman J. W. Carley D. W. (2009). Prevalence of type 2 diabetes in patients with obstructive sleep apnea in a multi-ethnic sample. J. Clin. Sleep. Med. 5 (3), 215–221. 10.5664/jcsm.27489 19960641 PMC2699165

[B55] Malhotra R. K. (2018). Neurodegenerative disorders and sleep. Sleep. Med. Clin. 13 (1), 63–70. 10.1016/j.jsmc.2017.09.006 29412984

[B56] Marciante A. B. Howard J. Kelly M. N. Santiago Moreno J. Allen L. L. Gonzalez-Rothi E. J. (2022). Dose-dependent phosphorylation of endogenous Tau by intermittent hypoxia in rat brain. J. Appl. Physiol. 133(3), 561–571. 10.1152/japplphysiol.00332.2022 35861520 PMC9448341

[B57] Marciante A. B. Wang L. A. Little J. T. Cunningham J. T. (2020). Caspase lesions of PVN-projecting MnPO neurons block the sustained component of CIH-induced hypertension in adult male rats. Am. J. Physiol. Heart Circ. Physiol. 318 (1), H34–H48. 10.1152/ajpheart.00350.2019 31675258 PMC6985804

[B58] Mortola J. P. (2007). Correlations between the circadian patterns of body temperature, metabolism and breathing in rats. Respir. Physiol. Neurobiol. 155 (2), 137–146. 10.1016/j.resp.2006.05.007 16815760

[B59] Mortola J. P. (1993). Hypoxic hypometabolism in mammals. Physiol. (Bethesda) 8 (2), 79–82. 10.1152/physiologyonline.1993.8.2.79

[B60] Mortola J. P. (2005). Influence of temperature on metabolism and breathing during mammalian ontogenesis. Respir. Physiol. Neurobiol. 149 (1-3), 155–164. 10.1016/j.resp.2005.01.012 16126013

[B61] Mortola J. P. Seifert E. L. (2002). Circadian patterns of breathing. Respir. Physiol. Neurobiol. 131 (1-2), 91–100. 10.1016/s1569-9048(02)00040-x 12106998

[B62] Mulkey D. K. Rosin D. L. West G. Takakura A. C. Moreira T. S. Bayliss D. A. (2007). Serotonergic neurons activate chemosensitive retrotrapezoid nucleus neurons by a pH-independent mechanism. J. Neurosci. 27 (51), 14128–14138. 10.1523/JNEUROSCI.4167-07.2007 18094252 PMC6673507

[B63] Mullins A. E. Kam K. Parekh A. Bubu O. M. Osorio R. S. Varga A. W. (2020). Obstructive sleep apnea and its treatment in aging: effects on alzheimer's disease biomarkers, cognition, brain structure and neurophysiology. Neurobiol. Dis. 145, 105054. 10.1016/j.nbd.2020.105054 32860945 PMC7572873

[B64] Nakamura A. Fukuda Y. Kuwaki T. (2003). Sleep apnea and effect of chemostimulation on breathing instability in mice. J. Appl. Physiol. 94(2), 525–532. 10.1152/japplphysiol.00226.2002 12433867

[B65] Orr J. E. Malhotra A. Sands S. A. (2017). Pathogenesis of central and complex sleep apnoea. Respirology 22 (1), 43–52. 10.1111/resp.12927 27797160 PMC5161664

[B66] Paxinos G. F. K. (2001). The mouse brain in stereotaxic coordinates. 2nd Edition. San Diego: Academic Press.

[B67] Peng Y. J. Zhang X. Gridina A. Chupikova I. McCormick D. L. Thomas R. J. (2017). Complementary roles of gasotransmitters CO and H2S in sleep apnea. Proc. Natl. Acad. Sci. U. S. A. 114 (6), 1413–1418. 10.1073/pnas.1620717114 28115703 PMC5307452

[B68] Phillipson E. A. (1978). Control of breathing during sleep. Am. Rev. Respir. Dis. 118 (5), 909–939. 10.1164/arrd.1978.118.5.909 216294

[B69] Piccione G. Caola G. Mortola J. P. (2005). Scaling the daily oscillations of breathing frequency and skin temperature in mammals. Comp. Biochem. Physiol. A Mol. Integr. Physiol. 140 (4), 477–486. 10.1016/j.cbpb.2005.02.010 15936708

[B70] Ramsden M. Kotilinek L. Forster C. Paulson J. McGowan E. SantaCruz K. (2005). Age-dependent neurofibrillary tangle formation, neuron loss, and memory impairment in a mouse model of human tauopathy (P301L). J. Neurosci. 25 (46), 10637–10647. 10.1523/JNEUROSCI.3279-05.2005 16291936 PMC6725849

[B71] Reznikov L. R. Meyerholz D. K. Abou Alaiwa M. Kuan S. P. Liao Y. J. Bormann N. L. (2018a). The vagal ganglia transcriptome identifies candidate therapeutics for airway hyperreactivity. Am. J. Physiol. Lung Cell Mol. Physiol. 315 (2), L133–L148. 10.1152/ajplung.00557.2017 29631359 PMC6139658

[B72] Reznikov L. R. Meyerholz D. K. Adam R. J. Abou Alaiwa M. Jaffer O. Michalski A. S. (2016). Acid-sensing ion channel 1a contributes to airway hyperreactivity in mice. PLoS One 11 (11), e0166089. 10.1371/journal.pone.0166089 27820848 PMC5098826

[B73] Reznikov L. R. Meyerholz D. K. Kuan S. P. Guevara M. V. Atanasova K. R. Abou Alaiwa M. H. (2018b). Solitary cholinergic stimulation induces airway hyperreactivity and transcription of distinct pro-inflammatory pathways. Lung 196 (2), 219–229. 10.1007/s00408-018-0091-0 29380034 PMC5975219

[B74] Santacruz K. Lewis J. Spires T. Paulson J. Kotilinek L. Ingelsson M. (2005). Tau suppression in a neurodegenerative mouse model improves memory function. Science 309 (5733), 476–481. 10.1126/science.1113694 16020737 PMC1574647

[B75] Schafer T. (2006). Respiratory pathophysiology: sleep-related breathing disorders. GMS Curr. Top. Otorhinolaryngol. Head. Neck Surg. 5, Doc01. 22073070 PMC3199805

[B76] Sheikhbahaei S. Gourine A. V. Smith J. C. (2017). Respiratory rhythm irregularity after carotid body denervation in rats. Respir. Physiol. Neurobiol. 246, 92–97. 10.1016/j.resp.2017.08.001 28782663 PMC5637156

[B77] Skatrud J. B. Dempsey J. A. (1983). Interaction of sleep state and chemical stimuli in sustaining rhythmic ventilation. J. Appl. Physiol. Respir. Environ. Exerc Physiol. 55 (3), 813–822. 10.1152/jappl.1983.55.3.813 6415011

[B78] Smith J. C. Ellenberger H. H. Ballanyi K. Richter D. W. Feldman J. L. (1991). Pre-botzinger complex: A brainstem region that may generate respiratory rhythm in mammals. Science 254 (5032), 726–729. 10.1126/science.1683005 1683005 PMC3209964

[B79] Speakman J. R. (2013). Measuring energy metabolism in the mouse - theoretical, practical, and analytical considerations. Front. Physiol. 4, 34. 10.3389/fphys.2013.00034 23504620 PMC3596737

[B80] Takaichi Y. Ano Y. Chambers J. K. Uchida K. Takashima A. Nakayama H. (2018). Deposition of phosphorylated alpha-synuclein in the rTg4510 mouse model of tauopathy. J. Neuropathol. Exp. Neurol. 77 (10), 920–928. 10.1093/jnen/nly070 30107539

[B81] Terwel D. Dewachter I. Van Leuven F. (2002). Axonal transport, tau protein, and neurodegeneration in Alzheimer's disease. Neuromolecular Med. 2 (2), 151–165. 10.1385/NMM:2:2:151 12428809

[B82] Trinder J. Ivens C. Kleiman J. Kleverlaan D. White D. P. (2006). The cardiorespiratory activation response at an arousal from sleep is independent of the level of CO(2). J. Sleep. Res. 15 (2), 174–182. 10.1111/j.1365-2869.2006.00513.x 16704573

[B83] Viemari J. C. Garcia A. J. 3rd Doi A. Ramirez J. M. (2011). Activation of alpha-2 noradrenergic receptors is critical for the generation of fictive eupnea and fictive gasping inspiratory activities in mammals *in vitro* . Eur. J. Neurosci. 33 (12), 2228–2237. 10.1111/j.1460-9568.2011.07706.x 21615559 PMC3652413

[B84] Yagishita S. Suzuki S. Yoshikawa K. Iida K. Hirata A. Suzuki M. (2017). Treatment of intermittent hypoxia increases phosphorylated tau in the hippocampus via biological processes common to aging. Mol. Brain 10 (1), 2. 10.1186/s13041-016-0282-7 28057021 PMC5217192

[B85] Yamauchi M. Ocak H. Dostal J. Jacono F. J. Loparo K. A. Strohl K. P. (2008). Post-sigh breathing behavior and spontaneous pauses in the C57BL/6J (B6) mouse. Respir. Physiol. Neurobiol. 162 (2), 117–125. 10.1016/j.resp.2008.05.003 18565803 PMC3698969

[B86] Zhang Q. Lai K. Xie J. Chen G. Zhong N. (2009). Does unrestrained single-chamber plethysmography provide a valid assessment of airway responsiveness in allergic BALB/c mice? Respir. Res. 10 (1), 61. 10.1186/1465-9921-10-61 19575792 PMC2719610

[B87] Zhu X. Zhao Y. (2018). Sleep-disordered breathing and the risk of cognitive decline: A meta-analysis of 19,940 participants. Sleep. Breath. 22 (1), 165–173. 10.1007/s11325-017-1562-x 28905231

